# TG6002 oncolytic vaccinia virus and chemotherapy synergy: a promising strategy for pancreatic ductal adenocarcinoma

**DOI:** 10.3389/fmicb.2025.1656984

**Published:** 2025-09-24

**Authors:** Johann Foloppe, Isabelle Farine, Christelle Pichon, Juliette Kempf, Julie Hortelano, Baptiste Moreau, Sandrine Cochin, Armelle Takeda, Patrick Pessaux, Philippe Erbs

**Affiliations:** ^1^Transgene, Illkirch-Graffenstaden, France; ^2^IHU, Strasbourg, France; ^3^Department of Visceral and Digestive Surgery, University Hospital of Strasbourg, Strasbourg, France

**Keywords:** pancreatic ductal adenocarcinoma, oncolytic vaccinia virus, TG6002, combination chemotherapy, patient-derived tumor models

## Abstract

Pancreatic ductal adenocarcinoma (PDAC) is a highly malignant cancer characterized by poor prognosis and limited treatment options, often associated with significant toxicity. Recently, we described TG6002, an oncolytic vaccinia virus (VACV) engineered for tumor-selective replication through the deletion of thymidine kinase and ribonucleotide reductase genes. TG6002 also expresses the suicide gene *FCU1*, which efficiently converts the non-cytotoxic prodrug 5-fluorocytosine (5-FC) into the chemotherapeutic agent 5-fluorouracil (5-FU). In this study, we evaluated the efficacy of TG6002, both alone and in combination with 5-FC, in human PDAC cell lines, xenograft and orthotopic PDAC models, as well as *ex vivo* in live patient-derived pancreatic cancer tissues. Additionally, we investigated the therapeutic potential of combining TG6002 with standard chemotherapeutic agents, including gemcitabine and components of the FOLFIRI regimen (irinotecan and oxaliplatin). *In vitro*, TG6002 demonstrated potent cytotoxicity against PDAC cells, which was further enhanced by the addition of 5-FC. Using a three-dimensional spheroid model, we confirmed TG6002’s ability to infect, replicate, and induce oncolysis. Furthermore, TG6002 replication was observed in surgically resected tumor tissues. Systemic administration of TG6002 showed significant antitumor activity in both subcutaneous and orthotopic PDAC models, with efficacy markedly improved by 5-FC administration. While TG6002 replication was inhibited by gemcitabine *in vitro*, TG6002 synergistically enhanced the cytotoxic effects of oxaliplatin and SN-38 (the active metabolite of irinotecan), primarily through increased apoptosis. In a xenograft model, systemic delivery of TG6002 with 5-FC, combined with either oxaliplatin or irinotecan, resulted in superior antitumor effects compared to monotherapy. In summary, our findings suggest that the systemic administration of TG6002 with 5-FC, in combination with irinotecan and oxaliplatin, represents a promising therapeutic strategy for PDAC patients.

## Introduction

Pancreatic ductal adenocarcinoma (PDAC) is the predominant form of pancreatic cancer. It is the seventh leading cause of cancer death worldwide and is projected to become a leading cause of cancer-related death in the coming years (Park et al., 2021). Pancreatic cancer is often detected at an advanced stage and has often spread to other parts of the body. PDAC is associated with an extremely poor prognosis, reaching an overall survival rate of less than 5% at 5 years and 24% at 1 year (Siegel et al., 2015). Even for those patients with resectable disease, the five-year survival rate is only 17% (Strobel et al., 2022). This poor prognosis is mainly due to the lack of early symptoms, rapid tumor progression and the limited efficacy of available treatments in metastatic disease (Lambert et al., 2019). Surgical resection with adjuvant chemotherapy remains the only treatment currently considered curative. However, only one-fifth of PDAC patients are diagnosed at a stage of surgically resectable localized disease, and relapse rates are high, exceeding 80%. For these patients, treatment consists of the current standard of care such as gemcitabine, more recently FOLFIRINOX (5-fluoruracil, leucovorin, irinotecan and oxaliplatin) or nab-paclitaxel, with only modest improvements in survival (Von Hoff et al., 2013; Kieler et al., 2020). The limitations of current treatments in pancreatic cancer underscore the need to explore new strategies to make significant therapeutic advances in this disease. The use of replication-competent viruses is an attractive strategy for tumor therapy. Oncolytic viruses are naturally occurring or engineered viruses that selectively infect and replicate in cancer cells, inducing oncolysis and antitumor immune responses. The targeting of these viruses to cancer cells is based on natural tropism and the deletion of viral genes required for replication in non-dividing cells. Several armed or unarmed oncolytic viruses, such as herpes simplex virus, adenovirus, attenuated measles virus, vesicular stomatitis virus and vaccinia virus (VACV), have been evaluated in preclinical studies and clinical trials in various indications (Zhu et al., 2023). And oncolytic viruses offer unique properties, making them an ideal choice for advanced PDAC tumors (Haller et al., 2020). We have developed TG6002, a highly attenuated oncolytic VACV with targeted deletions of two genes, thymidine kinase (TK; *J2R*) and a subunit of the ribonucleotide reductase (RR; *I4L*) to enhance tumor selective viral replication (Foloppe et al., 2019; Gallardo et al., 2020). In addition, TG6002 is armed with the chimeric yeast *FCU1* gene which encodes a bifunctional chimeric protein that catalyzes the conversion of 5-fluorocytosine (5-FC) into the toxic metabolites 5-fluorouracil (5-FU) and 5-fluorouridine monophosphate (5-FUMP) bypassing the natural resistance of tumor cells to 5-FU (Erbs et al., 2000). We have previously shown that tumor-specific expression of the *FCU1* gene by the virus induced high production of 5-FU in the tumor microenvironment (TME), by conversion of 5-FC, and an improved antitumor activity (Foloppe et al., 2019). Moreover, TG6002 induces an anti-tumor immune response involving CD8 T cells and tumor-infiltrating lymphocytes and myeloid cells (Heinrich et al., 2017). An open-label phase I dose-escalation trial of intravenous administration of TG6002 plus oral 5-FC was initiated in patients with advanced gastrointestinal malignancies (NCT03724071). Overall, the combination was well tolerated, and no maximum tolerated dose was observed. Preliminary results indicate effective biodistribution of TG6002 in tumor cells, associated with localized *FCU1* activity. In addition, a dose-escalation study of TG6002 via hepatic artery administration plus oral 5-FC was recently conducted in patients with liver-dominant metastatic colorectal cancer (NCT04194034). Intrahepatic artery delivery of the oncolytic virus was safe and this locoregional route of TG6002 administration targeted the tumor tissue and is associated with effective viral replication, expression and activity of the *FCU1* transgene, immune activation, and evidence of anti-tumor immune activity (West et al., 2025).

In the current study, we evaluated the therapeutic potential of TG6002 across various models of PDAC. Our investigation encompassed *in vitro* analyses using 2D and 3D cell cultures, *in vivo* assessments in xenograft and orthotopic models, and *ex vivo* studies on PDAC tumor biopsies. The findings demonstrate that TG6002 significantly reduces tumor growth and enhances survival. Additionally, we explored the combination of TG6002 with the current standard of care for pancreatic cancer. While gemcitabine was found to inhibit TG6002’s efficacy, synergistic interactions were observed with oxaliplatin and irinotecan, the key components of the FOLFIRINOX regimen. Collectively, these results support the potential application of TG6002 as a promising therapeutic option for patients with advanced PDAC, particularly those for whom effective standard treatments remain limited.

## Materials and methods

### Cell culture

Human PDAC cell lines AsPC-1 (CRL-1682™), BxPC-3 (CRL-1687™), Capan-2 (HTB-80™), MIA PaCa-2 (CRL-1420™) and PANC-1 (CRL-1469™), the normal human lung fibroblasts MRC-5 (CCL-171™) and the monkey kidney cell line Vero (CCL-81™) were obtained from the ATCC (Manassas, VA, United States). MIA PaCa-2, PANC-1 and Vero were grown in Dulbecco’s modified Eagle’s medium (DMEM) supplemented with 10% fetal calf serum (FCS). BxPC-3 and AsPC-1 were grown in Roswell Park Memorial Institute 1640 (RPMI 1640) medium supplemented with 10% FCS. Capan-2 was grown in McCoy’s 5a medium supplemented with 10% FCS. MRC-5 was grown in Eagle’s Minimum Essential Medium (EMEM) supplemented with 10% FCS. Primary chicken embryo fibroblasts (CEF) were used for recombination and amplification of viral vectors. CEF cells were prepared from chicken embryos derived from fertilized eggs (AVS Bio, Norwich, CT, United States) previously incubated for 11 or 12 days at 37°C in a humid atmosphere. Chicken embryos were dissected and treated with a 2.5% (w/v) solution of trypsin. CEF cells were maintained in Eagle-based Medium (MBE) supplemented with 5% FCS. All cells were incubated at 37°C in a humidified atmosphere containing 5% CO_2_.

### Drugs and chemicals

5-Fluorocytosine (Toronto Research Chemicals Inc., North York, Canada) and 5-Fluorouracil (Sigma-Aldrich, Saint-Louis, MO, United States) were solubilized in water and stored at −20°C. Oxaliplatin (Sigma-Aldrich) and SN-38 (Sigma-Aldrich) were solubilized in DMSO and stored at −20°C. Campto 20 mg/mL (Pfizer, Dublin, Ireland) were store at room temperature until use. Gemcitabin 40 mg/mL (Hexal AG, Holzkirchen, Germany) were stored at 4°C until use.

### Viruses

All oncolytic VACVs were derived from the Copenhagen strain and are deleted in thymidine kinase (*J2R*) and in the large subunit of RR (*I4L*) genes. TG6002 expressing the fusion gene *FCU1* (*ΔI4LΔJ2R*/FCU1 VACV) and VVTG17990 expressing *GFP* (*ΔI4LΔJ2R*/GFP VACV) were constructed and characterized previously (Foloppe et al., 2019; Beguin et al., 2020). Using the same methods, the double deleted VACV expressing luciferase (*ΔI4LΔJ2R*/LUC VACV), designated VVTG18277, was generated by homologous recombination using a shuttle plasmid encoding luciferase. VVTG18058 is a double deleted VACV without transgene (*ΔI4LΔJ2R* VACV). Viruses were amplified in CEF and purified, and virus stocks were titrated on Vero cells using plaque assay method.

### *In vitro* virus replication assay

Human PDAC cells were infected in suspension with TG6002 at MOI of 0.001. A total of 3 × 10^5^ cells/well were plated in 6-well culture plates and incubated in growth medium. Supernatants and cells were collected 24, 48 and 72 h post-infection and submitted to a quick freeze–thaw cycle and sonication to release intracellular viral particles. Viral titers in cell lysates were quantified on Vero cells by plaque assay.

### *In vitro* viral cytotoxicity assay

Human PDAC cells were infected in suspension by TG6002 at different MOIs. A total of 3 × 10^5^ cells/well were plated in 6-well culture plates in 2 mL of complete medium. The cells were then cultured at 37°C for 5 days and the viable cells were counted by trypan blue exclusion using a Vi-Cell Cell Counter (Beckman Coulter, Brea, CA, United States).

### *In vitro* FCU1 enzymatic assay

CDase and UPRTase activities in PDAC cell lines were determined using 5-Fluorocytosine (5-FC) and 5-Fluorouracil (5-FU) as substrates, respectively. PDAC cells (3 × 10^6^ cells) were infected by TG6002 and VVTG17990 at a MOI of 0.01. Twenty-four hours later, detection of CDase and UPRTase activities was performed by enzymatic assays as previously described (Erbs et al., 2008). Briefly, 5-FC, 5-FU and 5-FUMP were isocratically separated by HPLC (supelcosil LC-18-S column and UV detection at 260 nm and 280 nm). For detection of CDase activity, the mobile phase was 50 mM phosphoric acid adjusted to pH 2.1 with ammonium hydroxide. For detection of UPRTase activity, the mobile phase was 20 mM KH2PO4, 5 mM tetrabutylammonium sulfate, 5% methanol adjusted to pH5 with potassium hydroxide.

### *In vitro* cell sensitivity to 5-FC assay

Human PDAC cells were infected in suspension by the respective recombinant VACVs at a MOI that resulted in low cytotoxicity (MOI 10^−4^). A total of 3 × 10^5^ cells/well were plated in 6-well culture dishes in 2 mL of complete medium. At 48 h post-infection, cells were exposed to various concentrations of 5-FC for 3 days before determination of cell viability by a Vi-Cell Cell Counter. All samples were assayed in triplicate.

### Spheroid cultures

PANC-1 cell line was seeded at 2,000 cells per well in ultra-low attachment 96-well plates (Corning, Corning, NY, United States). To allow formation of spheroids, cells were incubated in DMEM containing 10% FCS for 4 days. To evaluate virus efficacy, spheroids were infected with varying amounts of VVTG17990 expressing GFP. Eight days after infection, spheroids were stained with propidium iodide (BD Biosciences, Franklin Lakes, NJ, USA) at 50 ng/well. Spheroids were imaged using the Celigo image cytometer (Nexcelom Biosciences, Lawrence, MA, United States). The diameter of each spheroid was measured using ImageJ software (National Institutes of Health, Bethesda, MD, United States) and converted to volume assuming a spherical shape. For replication assay, spheroids were infected at day four with TG6002 at 10, 100 and 1,000 PFU per well. At 1, 2, 3, and 4 days post-infection, spheroids and supernatants were harvested together and submitted to a quick freeze–thaw cycle and sonication. Viral titers were determined on Vero cells by plaque assay. For cell viability analysis, spheroids were infected at day four with TG6002 at 1, 10, 100, 1,000 and 10,000 PFU per well and cell viability was determined 8 days later by the CellTiter-Blue fluorescent assay (Promega Corporation, Madison, WI, United States) using the multi-wavelength automated Infinite M1000 plate-*reader* system (Tecan, Männedorf, Switzerland). The FCU1 activity was quantified by measuring the amount of 5-FC and 5-FU released in the culture media. Spheroids were infected at day 4 with TG6002 at 10 PFU and 0.3 mM 5-FC was added to the culture medium 24 h post-infection. Concentrations of 5-FC and 5-FU in the media were measured daily for 1 week by HPLC. Fifty μL of media were quenched with 50 μL of acetonitrile. Samples were vortexed and centrifuged. The organic supernatant was evaporated to dryness and reconstituted in 50 μL water and analyzed by HPLC using a mobile phase of 50 mM phosphoric acid adjusted to pH 2.1. Results are expressed as the percentage of 5-FU relative to the total amount of 5-FC + 5-FU after various incubation times with 5-FC.

### *In vitro* chemotherapy agent cytotoxicity assay

Human PDAC cells were plated in 6-well culture dishes in 2 mL of complete medium containing various drug concentrations. Cells were then cultured at 37°C for 5 days and the viable cells were counted by a Vi-Cell Cell Counter. All samples were assayed in triplicate.

### Combination of TG6002 with chemotherapies

Concentrations of TG6002 and chemotherapeutic drugs used in combitherapy assay were equal to IC50 for each cell culture. Human PDAC cells were infected in suspension with TG6002 at appropriate MOI and a total of 3 × 10^5^ cells/well were plated in 6-well culture dishes in 2 mL of complete medium containing the different chemotherapeutic agents at appropriate concentrations. The cells were then incubated at 37°C for several days. These combitherapy cultures were used to perform cytotoxicity assays and viral replication assays. Cell viability was evaluated after 5 days of treatment by a Vi-Cell Cell Counter. Viral titers were performed on cells and supernatants, 3 days post-infection. Viral titration was performed by plaque assay method on Vero cells after a rapid freeze–thaw cycle and sonication. All samples were assayed in triplicate. The effect of the combination of oncolytic Vaccinia virus and chemotherapeutic agents on cell proliferation was assessed by calculating combination-index (CI) values. Combination effects in PDAC cell lines were assessed by a constant ratio combination design in the range of 0.25 to 4-times the IC50 of the individual components. Human pancreatic cell lines were infected in suspension with various MOIs of TG6002. A total of 3 × 10^5^ cells/well were plated in 6-well culture dishes in 2 mL of medium supplemented with various concentrations of therapeutic agents. Cells were then incubated at 37°C for 5 days and the viable cells were counted by trypan blue exclusion. The mean CI was calculated from four data points at effective doses yielding 50, 75, 90 and 95% reduced viability, using the Compusyn program (ComboSyn Inc., Paramus, NJ, United States). This program applies the CI equation described by Chou and Talalay (Chou and Talalay, 1984). Combination index values below 0.9 were defined as synergism; between 0.9 and 1.1 as additive; and above 1.1 as antagonism. For apoptosis assay, after 24, 48 and 72 h of treatment, cells were trypsinized and were centrifuged at 300 g for 5 min to discard the supernatant and the trypsin. Cells were resuspended in PBS followed with addition of annexin V-FITC (Thermo Fisher Scientific, San Diego, CA, USA) and incubated for 30 min at room temperature. Cells were washed with PBS and centrifuged at 300 g for 5 min and resuspended in 500 μL PBS followed by addition of propidium iodide (PI) addition (Thermo Fisher Scientific). After staining, cells were analyzed by flow cytometry on MACSquant Analyzer 10 Flow Cytometer (Miltenyi Biotec, Bergisch Gladbach, Germany) and analyzed using Kaluza software 2.1 (Beckman Coulter). Cells were classified according to annexin V-FITC and PI labeling as viable (double negative), early apoptotic (annexin V-FITC single-stained cells), necrotic cells (PI single-stained cells) and cells in advanced phases of apoptosis (double-stained cells).

### Animals

Animal experiments were approved by the local regulatory authority (Comité d’éthique en matière d’expérimentation animale de *Strasbourg, France*) and conducted in accordance with European and French legislations on the protection of animals used for scientific purposes.

### Subcutaneous tumor models

Female Swiss nude mice were purchased from Charles River Laboratories (Saint Germain Nuelles, France). Animals used in the studies were uniform in age (6 weeks) and body weight (20–23 g). Mice were injected subcutaneously (s.c.) into the right flank with 5 × 10^6^ MIA PaCa-2 cells. When tumors reached a diameter of 100–200 mm^3^, the mice were randomized in a blinded manner and treated with the indicated vectors.

### Orthotopic pancreatic model

Swiss nude mice were anesthetized by inhalational isofurane (Iso-Vet, Piramal Critical Care, Bethlehem, United States) and subcutaneous injection of xylazine (Rompun 2%, Elanco Animal Health, Greenfield, IN, United States) and buprenorphine (Vetergesic, Ceva Animal Health, Libourne, France). After local shaving and disinfection, the abdominal skin and muscle were incised just off the midline and directly over the pancreas to allow visualization of the pancreatic lobes. The pancreas was positioned for direct injection of a 20 μL of 2 × 10^5^ MIA PaCa-2 using a 27-gauge needle and calibrated syringe. To further prevent leakage, the needle was held on the injection site for 30 s prior to removal. The pancreas was then returned within the abdominal cavity and both the muscle and skin layers were closed with surgical glue. Following recovery from surgery, the mice were monitored and weighed daily.

### Bioluminescence imaging

In the orthotopic model and 50 days after tumor implantation, VVTG18277 expressing luciferase was injected IV at 1 × 10^7^ PFU. Bioluminescence imaging was performed 4, 7 and 11 days post-virus injection. Prior to imaging, Swiss nude mice were anesthetized under isoflurane gas and were injected intraperitoneally with 200 μL of 15 mg/mL D-luciferin (Xenogen Corporation, Alameda, CA, United States). After 10 min, luminescence imaging was performed using the IVIS lumina II (Caliper Life Sciences, Hopkinton, MA, USA). Data acquisition and analysis were performed using Living Image software 2.5 (Caliper Life Sciences).

### Antitumor activity of TG6002 in subcutaneous pancreatic model

To analyze anti-tumor efficacy after IT delivery, TG6002 was injected once into the tumors at 1 × 10^3^, 1 × 10^4^, 1 × 10^5^ or 1 × 10^6^ PFU. For IV treatment, mice received 1 × 10^7^ PFU of TG6002 *via* tail-vein injection and starting day 5 following viral injection, the animals were treated with *per os* administrations of saline or 5-FC (200 mg/kg/day) twice daily for 3 weeks. Tumor size was measured twice a week using calipers. Tumor volumes were calculated in mm^3^ using the formula (*Π*/6) (length x width^2^).

### Antitumor activity of TG6002 in orthotopic pancreatic model

For IT administration, the abdominal cavity of the mice was opened under anesthesia 36 days after implantation and 1 × 10^7^ PFU of TG6002 was injected into the tumors in a volume of 100 μL. The operative wound was subsequently closed with a longitudinal suture. For IV administration, TG6002 was injected once by tail vein injection at 1 × 10^7^ PFU and starting day 7 following viral injection, the animals were treated with *per os* administrations of saline or 5-FC (200 mg/kg/day) twice daily for 3 weeks. Animal survival was monitored by assessment of animal well-being by monitoring weight (terminal weight loss considered at 20% of original body weight), clinical signs (reduced motion, ruled fur, no response to external stimuli) and ascites formation (massive ascites with abdominal circumference greater than 66 mm).

### Antitumor activity of the combination of TG6002/5-FC and chemotherapies in subcutaneous models

TG6002 was injected intravenously three times at 1 × 10^6^ PFU, 3 days apart. Starting 3 days after the first virus injection, mice were then treated intravenously by irinotecan (33 mg/kg/day) or intraperitoneally by oxaliplatin (2.5 mg/kg/day) twice weekly for 3 weeks. Mice were also treated with *per os* administrations of saline or 5-FC (200 mg/kg/day), twice daily starting on day 4 post last virus injection for 3 weeks. Tumor size was measured twice a week.

### Precision-cut tissue slices from surgical specimens

The study was approved by the Ethics Committee of IHU/IRCAD (Strasbourg, France), and written informed consent was obtained from the patient prior to surgery. Under the direction of the surgeons in consultation with Pathology staff, fresh biopsy samples of pancreatic tumor and non-tumoral adjacent tissue (duodenum) were collected immediately after surgical resection. Tissues were transported and kept on ice until sectioning. The tissue pieces were sectioned using the vibratome VT1200s (Leica Biosystems, Nussloch, Germany). Tissues were cut into 300 μm thick slices with following operational settings: speed 0.4 mm/s, amplitude 1.4–1.8 mm, blade angle of 15°. Slices were initially collected in PBS supplemented with 100 U/mL penicillin and 100 mg/mL streptomycin. Using a thin brush, the tissue slices were transferred to 24-well plates containing 500 μL of DMEM/F12 medium, 20% fetal bovine serum, 2 mM glutamine, 100 U/mL penicillin, 100 mg/mL streptomycin and 40 mg/mL gentamycin. Subsequently, tissue slices were treated with 1 × 10^6^ PFU of TG6002. Mock-treated slices served as negative control. Tissue slices were incubated at 37°C in a 5% CO_2_ atmosphere. After 16 h incubation (to allow viral adsorption), tissue slices were washed with PBS and recultured in fresh media. After 3 days of culture, tissue slices were fixed in 4% paraformaldehyde for 24 h and immunohistochemistry was performed using a rabbit anti-VACV polyclonal antibody (1:1400 dilution) [Meridian Life Science, catalog code (B65101R), Memphis, TN, United States]. Immunohistochemical analyses were performed on BOND RX^m^ automated system (Leica BioSystems) using Novolink Polymer Detection Systems method (Leica BioSystems), fluorescence revelation and Hoechst counterstaining on formalin-fixed tissue section.

### Statistical analysis

*In vitro*, data were analyzed by one-way analysis of variance (ANOVA) followed by post-hoc tests using Tukey adjustment. Statistical analyses of tumor volume growth were performed, using a mixed model to take the longitudinal aspect into account (Foloppe et al., 2019). The inter-individual heterogeneity was considered as a random effect in the model. Tumor sizes were converted in equivalent diameters to comply with the mixed model assumption and to get a better fit to the statistical model (Bastogne et al., 2010). Comparisons between treatments were also performed using least-squares means. To study non-inferiority relations, overall estimation of tumor size growth with the associated interval of measurement uncertainty was compared. Survival analyses in the orthotopic pancreatic model were performed using log-rank test. Statistical analyses were performed using R software. Differences were considered statistically significant at p < 0.05, with significance levels denoted as follows: *p* < 0.05 (*), *p* < 0.01 (**), *p* < 0.001 (***), and *p* < 0.0005 (****).

## Results

### *In vitro* oncolytic activity of TG6002 against PDAC cells

**Figure 1:**
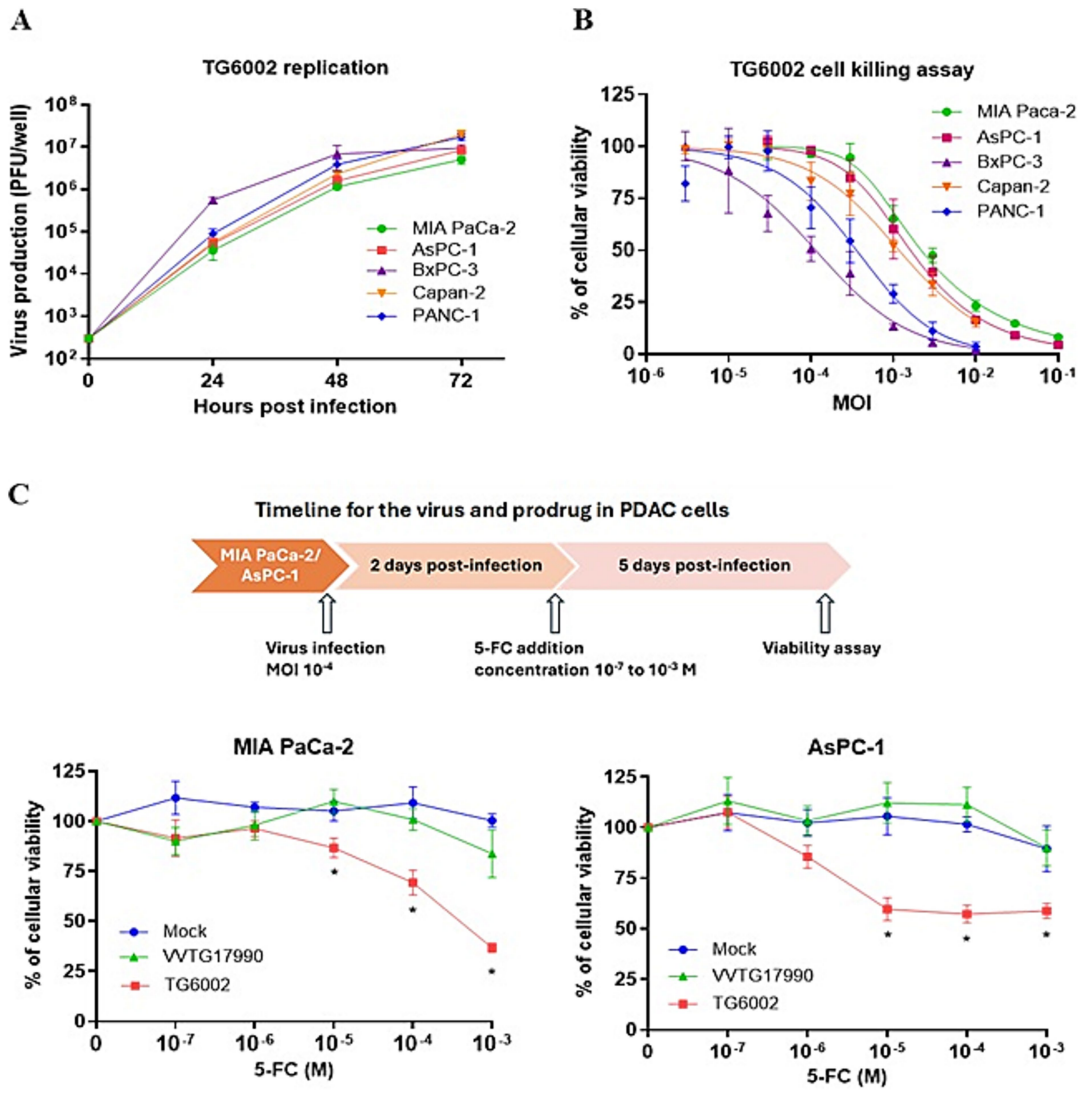
*In vitro* characterization of replication and therapeutic activity of TG6002 on human PDAC cells using 2D Monolayer Cultures. **(A)** Multistep growth curves of TG6002 at different time points from a panel of human PDAC cells when infected at MOI 0.001. The results are presented as a mean of triplicate experiments ± SD. **(B)** Dose–response analysis of TG6002 in PDAC cells. Cell viability was determined 5 days post-infection by trypan blue exclusion. The results are presented as a mean of triplicate experiments ± SD. **(C)** Combination of oncolytic and prodrug cytotoxicity in the 2 most resistant PDAC cell lines to virus oncolytic activity. MiA PaCa-2 and AsPC-1 cells were infected at a MOI of 10^−4^ with TG6002. After 48 h, 5-FC was added in a range of concentrations, and cell survival was determined 3 days later by trypan blue exclusion. Results were standardized against values for wells non-treated, lacking virus and drug, which represented 100% viability. Values are represented as means ± SD of three individual determinations.

**Figure 2:**
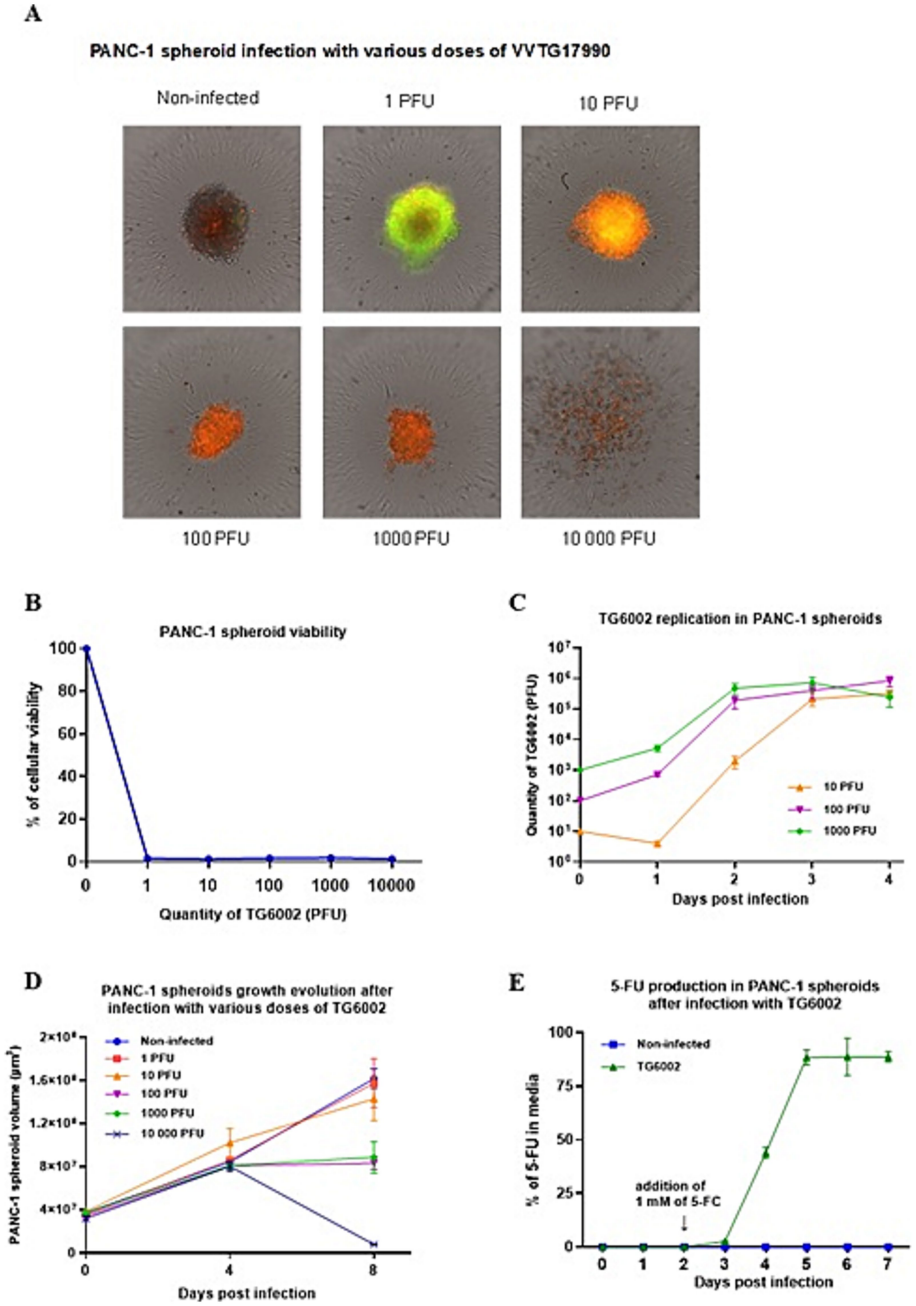
*In vitro* characterization of replication and therapeutic activity of TG6002 on a PDAC cell in 3D culture model. **(A)** PANC-1 spheroid infection and necrosis induced by VVTG17990. PANC-1 spheroids were infected at indicated doses of VVTG17990 at D4. Eight days after infection, spheroids were treated with 50 ng propidium iodide/well, GFP expression and propidium iodide staining were observed and confirm the susceptibility of PANC-1 spheroids to VACV infection and killing capacity. **(B)** TG6002 oncolytic activity on spheroids. PANC-1 spheroids were infected with the indicate doses of TG6002 at D4 and cell viability was determined at D8 using the CellTiter-Blue fluorescent assay. Each data point represents the mean of triplicate determinations ± SD. **(C)** Viral replication kinetics of TG6002 on PANC-1 spheroids. Replication capacity was analyzed through viral growth curve by recovering infected PANC-1 spheroids (10, 100 and 1,000 PFUs per well) at different time points post-infection. The titers of progeny virus were determined by plaque assay. The results are presented as a mean of triplicate experiments ± SD. **(D)** Growth of spheroids after infection. PANC-1 spheroids were infected with the indicate doses of TG6002 at D4 and spheroid volume was determined at D8 and D12 as described in Materials and Methods. Each data point represents the mean of triplicate determinations ± SD. **(E)** FCU1 activity in spheroid. Spheroids were infected with 10 PFU of TG6002 at D4. After 48 h, 5-FC was added at 1 mM, and each day from D3 to D7 after infection, the percentage of 5-FU produced in the supernatant was determined as described in Materials and Methods. Values are represented as means ± SD of three individual determinations.

The replicative and cytolytic capacities of TG6002 were initially evaluated in 2D monolayer cultures of human PDAC cell lines, representing epithelial (AsPC-1, BxPC-3, Capan-2) and mesenchymal (MIA PaCa-2, PANC-1) differentiation subtypes (Dijk et al., 2020). Viral replication was assessed using a multistep growth assay, where PDAC cells were infected with TG6002 at a MOI of 10^−3^, and virus production was measured at 24, 48, and 72 h post-infection. High replication levels were observed, with a 100- to 1,000-fold increase in viral particles at 24 h and over 10,000-fold at 72 h post-infection. Among the cell lines, BxPC-3 demonstrated the highest sensitivity, rapidly producing large quantities of the virus (Figure 1A). The oncolytic activity of TG6002 was evaluated by infecting PDAC cells across a range of MOIs and assessing cell viability 5 days post-infection. TG6002 exhibited cytotoxicity at relatively low MOIs, with half-maximal effective concentrations (EC50) below an MOI of 10^−2^ in all tested cell lines. BxPC-3 and PANC-1 were the most susceptible, with viability dropping below 50% at an MOI of 10^−3^ (Figure 1B). These findings revealed a correlation between viral replication efficiency and cytolytic activity. The permissiveness ranking of PDAC cell lines for TG6002 was consistent across both assays: BxPC-3 > PANC-1 > Capan-2 > AsPC-1 > MIA PaCa-2. The expression of the *FCU1* transgene was analyzed by measuring the enzymatic activities of cytosine deaminase (CDase) and uracil phosphoribosyltransferase (UPRTase), which mediate the conversion of 5-FC to 5-FU and 5-FU to 5-FUMP, respectively. One day post-infection, TG6002-infected PDAC cells displayed significant CDase and UPRTase activity, while mock-infected or non-armed VACV (VVTG18058)-infected cells showed none (Table 1). This confirmed the production of functional FCU1 protein. Since MIA PaCa-2 and AsPC-1 cells were the least sensitive to TG6002-mediated oncolysis (Figure 1B), we assessed the impact of prodrug conversion on cytotoxicity. These cell lines were infected with VVTG17990 or TG6002 at a low MOI (10^−4^) and treated with 5-FC at different concentrations. Without 5-FC, no cytotoxicity was observed at this low MOI. As expected, 5-FC addition did not increase cytotoxicity in cells infected with VVTG17990. However, in TG6002-infected cells, 5-FC induced significant, dose-dependent cytotoxicity (Figure 1C). This confirmed that TG6002-mediated FCU1 expression enabled effective prodrug activation and tumor cell killing. The therapeutic potential of TG6002 was further investigated in a 3D culture system using PANC-1 spheroids grown in ultra-low attachment plates. Spheroids were infected with VACV expressing GFP at MOIs ranging from 1 to 10,000 PFU. At day 8 post-infection, GFP fluorescence indicated extensive viral spread within the spheroids, even at 1 PFU. At higher MOIs (10–1,000 PFU), significant cell death, as evidenced by propidium iodide staining, was observed, with complete spheroid disaggregation occurring at 10,000 PFU (Figure 2A). Cell viability, assessed using the CellTiter-Blue assay, confirmed that VACV induced cytotoxicity even at the lowest dose (Figure 2B). Viral replication kinetics over 4 days showed significant amplification, plateauing at 5 × 10^5^ PFU regardless of the initial dose (Figure 2C). Spheroid volume measurements revealed dose-dependent growth inhibition 8 days post-infection (Figure 2D). Additionally, TG6002’s ability to convert 5-FC to 5-FU via FCU1 expression was confirmed in 3D cultures, with complete 5-FC conversion detected 3 days after its addition to TG6002-infected spheroids (Figure 2E). Collectively, these results demonstrate the functionality and therapeutic potential of TG6002 in both 2D and 3D culture systems.

**Table 1:** Specific CDase and UPRTase activities.

Cell lines	Virus	CDase 5-FC → 5-FU	UPRTase 5-FU → 5-FUMP
AsPC-1	Mock	ND	ND
	VVTG18058	ND	ND
	TG6002	359.6 ± 22.1	99.4 ± 12.1
Capan-2	Mock	ND	ND
	VVTG18058	ND	ND
	TG6002	315.1 ± 15.3	84.4 ± 7.3
MIA PaCa-2	Mock	ND	ND
	VVTG18058	ND	ND
	TG6002	383.6 ± 33.6	102.7 ± 14.7

PDAC cells were infected at MOI 0.01 with the indicated vectors. After 24 h, enzymatic activities were measured as described in Materials and Methods. CDase and UPRTase activities are expressed as the number of nanomole of 5-FC deaminated per min per mg of protein and the number of nanomole of 5-FU phosphorylated per min per mg of protein, respectively. The indicated enzymatic activities were measured as described in the Materials and Methods. Each value represents the average of three independent experiments ± SD. CDase, cytosine deaminase; 5-FC, 5-fluorocytosine; 5-FU, 5-fluorouracil; 5-FUMP, 5-fluorouridine-5′-monophosphate; ND, not detectable; UPRTase, uracil phosphoribosyltransferase.

### *In vitro* combination of TG6002 with chemotherapy for PDAC

**Figure 3:**
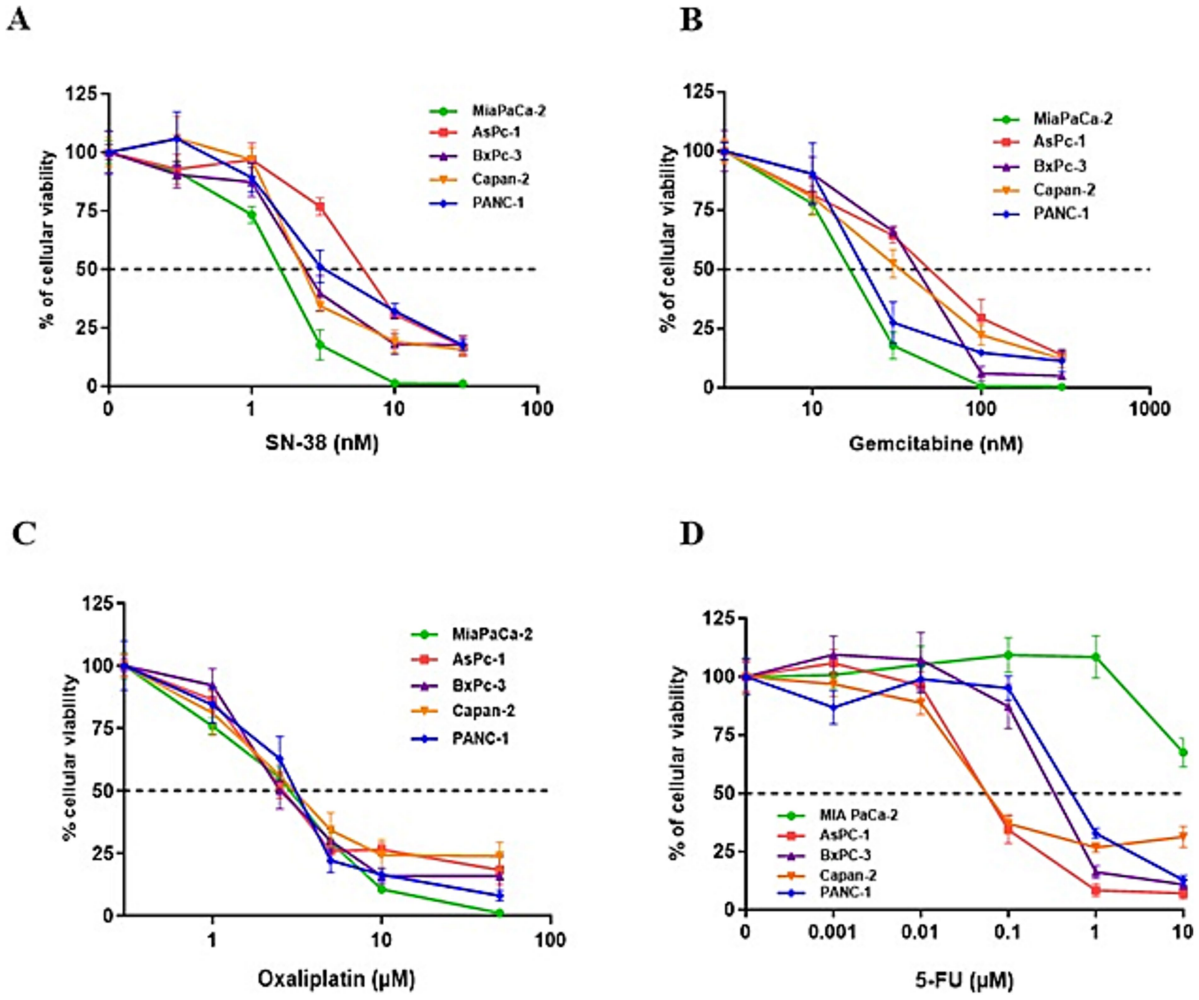
Treatment of PDAC cells with different standard chemotherapies. PDAC cell lines were treated for 5 days with increasing concentrations of SN-38 **(A)**, gemcitabine **(B)**, oxaliplatin **(C)**, or 5-FU **(D)**. Cell viability was assessed using a Vi-CELL automated cell counter based on the Trypan Blue exclusion method. Each data point represents the mean ± SD of triplicate measurements. The dotted line indicates the drug concentration required to reduce cell viability by 50% (IC50).

**Figure 4:**
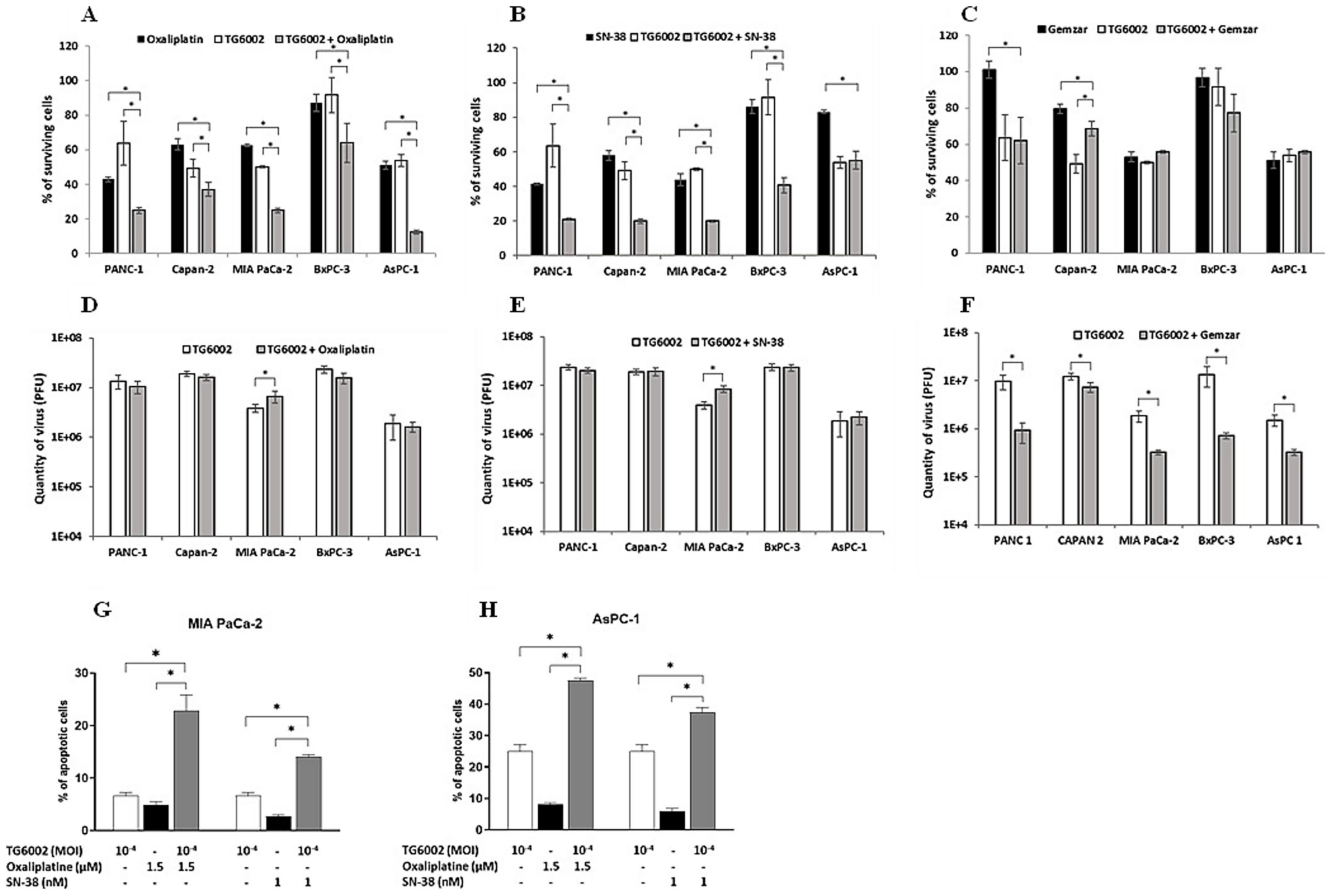
Effect of combination of TG6002 with chemotherapeutic agents on cell killing activity, TG6002 replication and apoptosis induction in PDAC cells. Cell lines were infected by TG6002 and treated by chemotherapeutic agents. Cell viability was determined 5 days after infection and/or treatment with oxaliplatin **(A)**, SN38 **(B)** or gemcitabine **(C)** using trypan blue exclusion method. Results are presented as the percentage of remaining cell. Virus titer determination was performed 72 hrs after infection/treatment with oxaliplatin **(D)**, SN38 **(E)** or gemcitabine **(F)** using plaque assay method. The concentrations used for each cell line were indicated in Table 2. MIA PaCa-2 **(G)** and AsPC-1 **(H)** PDAC tumor cell lines were treated with therapeutic concentrations of oxaliplatin (1.5 µM) or SN-38 (1 nM) or/and TG6002 at MOI 10-4 during 4 days. Percentage of apoptotic cells was determined by flow cytometry after cell staining with Annexin V and propidium iodide. All results are represented as mean of triplicate determinations ± SD. * *p* < 0.05 (one-way ANOVA).

We next assessed the antiproliferative effects of the standard chemotherapeutic agents used in pancreatic cancer treatment—namely gemcitabine, 5-FU, oxaliplatin, and SN-38 (the active metabolite of irinotecan)—on PDAC cell lines. The cell lines were exposed to increasing concentrations of these agents, revealing varying levels of susceptibility. Among the tested drugs, SN-38 and gemcitabine were the most effective, with half-maximal inhibitory concentrations (IC_50_) of approximately 3 nM and 30 nM, respectively. MIA PaCa-2 cells were the most sensitive to these agents, while AsPc-1 cells were the most resistant (Figures 3A,B). Oxaliplatin showed similar sensitivity across all five PDAC cell lines, with an IC50 of ~3 μM (Figure 3C). In contrast, 5-FU exhibited the highest heterogeneity, with IC50 values ranging from 10 μM in the highly resistant MIA PaCa-2 cells to <0.1 μM in the highly sensitive Capan-2 and AsPc-1 cells (Figure 3D). We investigated the cytotoxic effects of combining TG6002 with chemotherapeutic agents. PDAC cells were treated with oxaliplatin, SN-38, or gemcitabine alone, TG6002 alone, or a combination of TG6002 with each chemotherapeutic agent (Figures 4A–C). Treatments were administered concurrently at concentrations approximating the IC₅₀ values for each agent (Table 2). The combination of TG6002 with oxaliplatin significantly enhanced cytotoxicity across all tested PDAC cell lines (Figure 4A). For example, in AsPc-1 cells, co-treatment with 5 μM oxaliplatin and TG6002 at a multiplicity of infection (MOI) of 10^−4^ induced 90% cell death, compared to 50% with either agent alone. Similarly, combining TG6002 with SN-38 produced significantly greater cytotoxicity than either treatment alone (Figure 4B). For example, in BxPc-3 cells, TG6002 (MOI 10^−6^) or SN-38 (1 nM) alone reduced cell viability to 90 and 85%, respectively, while their combination decreased viability to 40%. However, combining TG6002 with gemcitabine did not improve cytotoxicity over TG6002 or gemcitabine alone. (Figure 4C). We next measured the impact of the chemotherapeutic agents on the production of TG6002 on PDAC cells (Figures 4D–F), using the same concentrations as in the cytotoxicity assays (Table 2). Oxaliplatin and SN-38 had minimal or no impact on TG6002 replication (Figures 4D,E), suggesting that the enhanced cytotoxicity observed with these combinations was not due to improved viral replication. In contrast, gemcitabine appeared to inhibit TG6002 efficacy, as viral replication was significantly reduced in its presence. Viral particle production decreased by 2- to 100-fold depending on the cell line (Figure 4F). To further understand the synergistic interactions between TG6002 and SN-38 or oxaliplatin, we assessed apoptosis rates using Annexin V/Propidium Iodide double staining and flow cytometry (Figures 4G,H). The doses used were sub-cytotoxic to isolate potential synergistic effects. Combination treatments dramatically increased apoptosis rates in both MIA PaCa-2 (Figure 4G) and AsPc-1 cells (Figure 4H), consistent with the cytotoxicity data (Figures 4A,B). Finally, we calculated the combination index (CI) using isobologram analysis to quantify synergy. The mean CI values ranged from 0.45 to 1 across the five PDAC cell lines, indicating moderate to strong synergy (Table 3). The most potent synergistic response was seen with SN-38 in Capan-2 cells where simultaneous TG6002 infection and SN-38 addition resulted in CI value <0.5. Strong synergy was also observed with oxaliplatin and SN-38 in MIA PaCa-2 cells, with CI values <0.6. For both drugs, the lowest degree of synergistic interaction was observed in MIA PaCa-2 cells, with CI values approximating 1, indicative of an additive effect (Table 3). In summary, the combination of TG6002 with either oxaliplatin or SN-38 markedly enhanced tumor cell death, primarily by promoting apoptosis, without impairing viral replication. In contrast, gemcitabine inhibited TG6002 replication, thereby diminishing the effectiveness of the combination therapy.

**Table 2:** Concentration of TG6002, oxaliplatin, SN-38 and gemcitabine used in combination assay

Cell lines	TG6002 (MOI)	Oxaliplatin (μM)	SN-38 (nM)	Gemcitabine (nM)
PANC-1	10^-5^	3	3	10
Capan-2	10^-5^	5	2	50
MIA PaCa-2	10^-4^	3	2	20
BxPC-3	10^-6^	1	1	10
AsPC-1	10^-4^	5	3	50

The specified concentrations were employed in the cell killing and replication assays presented in Figures 4A–F. PDAC cells were infected with TG6002 at appropriate MOI and treated with appropriate concentrations of the different chemotherapeutic agents for cell killing assays or viral replication analysis.

**Table 3:** Combination index for the combination TG6002 with oxaliplatin or SN-38 in PDAC cell lines.

	AsPC-1	BxPC-3	Capan-2	MIA PaCa-2	PANC-1
Oxaliplatin	0.80	0.88	0.70	0.51	0.99
SN-38	0.88	0.69	0.45	0.55	1.04

PDAC tumor cell lines were treated with increasing concentrations of TG6002 and oxaliplatin or SN-38, alone or in combination. Cell viability was determined by ViCell cell counter automate based on trypan blue exclusion method. The Mean CI was calculated by an average of the CI obtained at each fraction affected given by CompuSyn software. CI values below 0.9 were defined as synergism; between 0.9 and 1.1 as additive; and above 1.1 as antagonism.

### Antitumor activity of TG6002 in ectopic and orthotopic pancreatic tumor models

**Figure 5:**
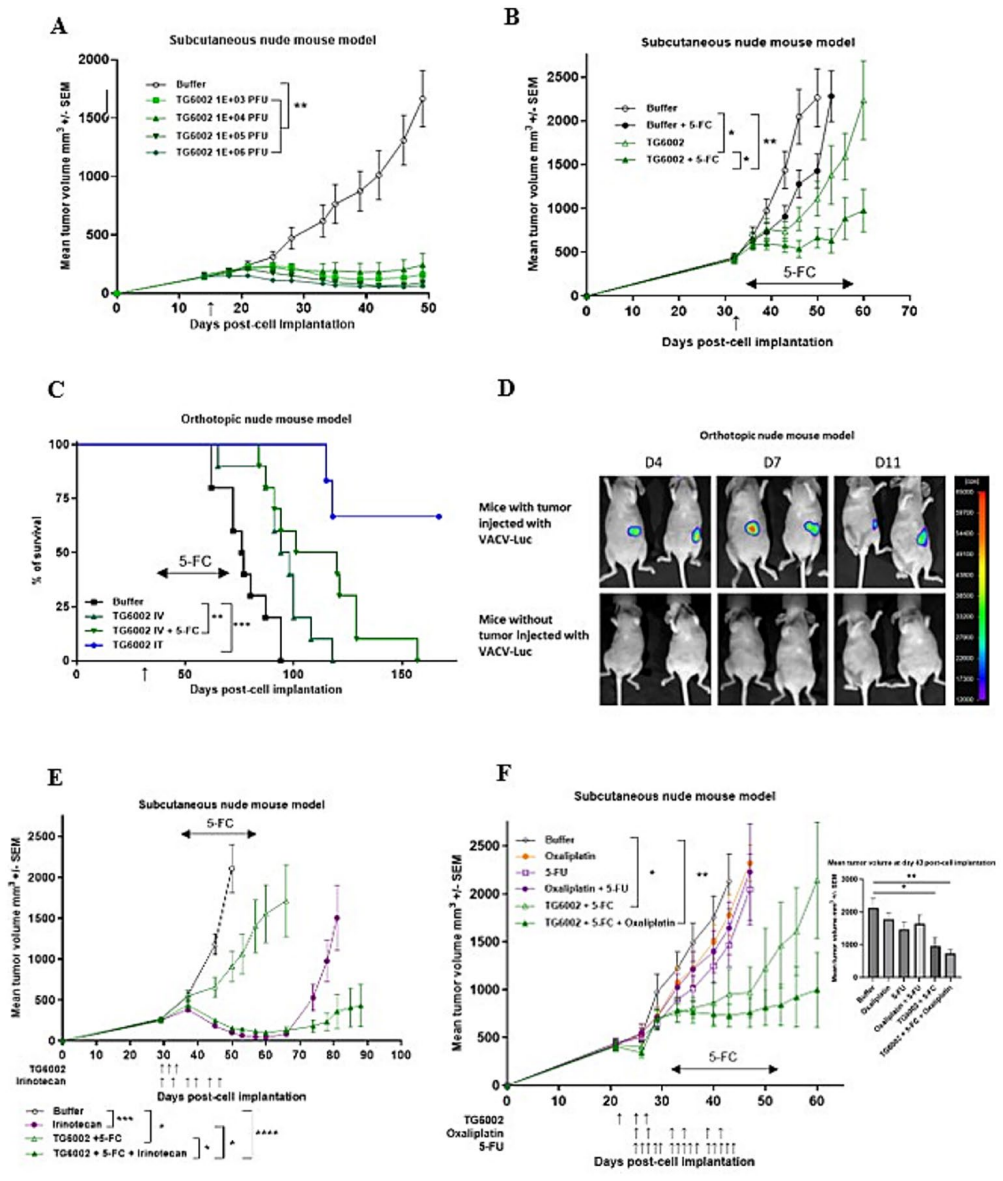
Antitumor activity of TG6002 alone or in combination in human pancreatic tumor models. **(A)** Antitumor activity of TG6002 after i.t. injection in a subcutaneous Mia-PACA-2 model. TG6002 was injected at different doses 15 days after cell implantation (indicated by a vertical arrow). Tumors were measured in three dimensions and represented as mean ± SEM (*n* = 10). **(B)** Antitumor activity of TG6002 after i.v. injection ± 5-FC in a subcutaneous Mia-PACA-2 model. TG6002 was injected at 1x10^7^ PFU 32 days after cell implantation (indicated by a vertical arrow). Five days after viral injection, mice were treated twice daily with *per os* administrations of saline or 5-FC (200 mg/kg/day) for 3 weeks (indicated by a horizontal arrow). Tumors were measured in three dimensions and represented as mean ± SEM (*n* = 10). **(C)** Antitumor activity of TG6002 in an orthotopic human pancreatic tumor model. MIA PaCa-2 cells were injected into the pancreas of nude mice. 36 days after cell implantation, TG6002 was injected i.v. or i.t. at 1x10^7^ PFU (indicated by a vertical arrow). Seven days after viral injection (i.v.), mice were treated twice daily with *per os* administrations of saline or 5-FC (200 mg/kg/day) for 3 weeks (indicated by a horizontal arrow). Percentage of mice survival was represented (n=8). **(D)** To monitor viral distribution, bioluminescence imaging was performed in mice bearing orthotopic pancreatic tumors and in tumor-free control mice. Animals received an intravenous injection of 1×10^7^ PFU of VVTG18277 (VACV-Luc), and luciferase activity was assessed at days 4, 7, and 11 post-injection. Representative luminescence images are shown for both tumor-bearing (top panel) and non-tumor-bearing mice (bottom panel), illustrating virus-dependent luciferase expression. **(E)** Antitumor activity of the combination of TG6002 and irinotecan in a subcutaneous model. TG6002 was injected i.v. in nude mice bearing subcutaneous pancreatic MIA-PaCa-2 human tumors. Virus was injected three times at 1x10^6^ PFU at days 30, 32 and 34 after cell implantation. The animals were treated by irinotecan twice per week (33 mg/kg/day) for 3 weeks from day 30 (indicated by vertical arrows). The mice were also treated twice daily with *per os* administrations of saline or 5-FC (200 mg/kg/day) for 3 weeks from day 36 (indicated by a horizontal arrow). Tumors were measured in three dimensions and represented as mean ± SEM (*n* = 10). **(F)** Antitumor activity of the combination of TG6002 and oxaliplatin in subcutaneous models. TG6002 was injected i.v. in nude mice bearing subcutaneous pancreatic MIA-PaCa-2 tumors. Virus was injected three times at 1x10^6^ PFU at days 22, 25 and 27 after cell implantation. The animals were then treated by 5-fluorouracil 5 times a week (20 mg/kg/day) for 3 weeks from day 25 or twice a week by oxaliplatin (2.5 mg/kg/day) for 3 weeks from day 25 (indicated by vertical arrows). The mice were also treated twice daily with *per os* administrations of saline or 5-FC (200 mg/kg/day) for 3 weeks from day 32 (indicated by a horizontal arrow). Tumors were measured in three dimensions and represented as mean ± SEM (*n* = 10). Bar graph represents the tumor volumes 43 days post-cell implantation. For tumor volume analysis, statistical significance was determined using a mixed model to take the longitudinal aspect into account. For survival analyses, statistical significance was determined using the log-rank test. * *p* < 0.05, ** *p* < 0.01, *** *p* < 0.001, **** *p* < 0.0005.

We assessed the oncolytic activity of TG6002 in a subcutaneous MIA PaCa-2 model. TG6002 was administered either via intratumoral (i.t.) injections with escalating doses ranging from 1 × 10^3^ to 1 × 10^6^ PFU (Figure 5A) or as a single intravenous (i.v.) injection at 1 × 10^7^ PFU (Figure 5B). In the i.v. experiment, 5-FC was administered orally for 3 weeks starting on day 7 post-viral delivery. Regardless of the i.t. dose, tumor growth was significantly suppressed (Figure 5A). Tumor volume regression was observed across all treated groups (*p* < 0.01 compared to the control group), with the timing of regression correlating to the viral dose administered. The earliest regression was seen in the group receiving the highest i.t. dose of TG6002 (1 × 10^6^ PFU; Figure 5A). A single intravenous injection of TG6002 significantly delayed tumor growth compared to the control group (*p* < 0.05; Figure 5B). Administration of 5-FC alone did not affect tumor growth; however, 5-FC significantly enhanced the antitumor activity of TG6002 compared to the virus alone (*p* < 0.05) and to the control group (*p* < 0.01). This finding indicates that the FCU1/5-FC system compensates for the relatively modest oncolytic activity of TG6002 in this model. To further evaluate antitumor efficacy, the virus was administered either intratumorally or intravenously in mice bearing orthotopic pancreatic tumors generated by transplantation of MIA PaCa-2 cells. (Figure 5C). Mice were euthanized upon reaching predefined endpoints, including significant weight loss, clinical signs, and ascites formation, as detailed in the Materials and Methods section. The i.v. injection of TG6002 (1 × 10^7^ PFU) improved survival compared to controls, and this effect was significantly enhanced by 5-FC treatment (*p* < 0.01 compared to the control group). The i.t. injection of the same TG6002 dose resulted in the highest survival rate with more than 60% of survival (*p* < 0.001 compared to the control group; Figure 5C). To assess viral replication in the orthotopic model, a TK- and RR-deleted vaccinia virus expressing luciferase (VACV-Luc) was administered intravenously at 1×10^7^ PFU. Bioluminescence imaging performed at days 4, 7, and 11 post-infection revealed luciferase signals exclusively in the pancreas of tumor-bearing mice, confirming tumor-specific viral replication (Figure 5D). Strong bioluminescent signals were localized to the tumor site, indicating active viral replication. In contrast, no luciferase signal was detected in control mice without tumors, demonstrating the absence of viral replication in non-tumor tissue. In a second set of in vivo experiments, we evaluated the combination of TG6002/5-FC with standard-of-care (SOC) chemotherapeutic agents for pancreatic cancer, irinotecan and oxaliplatin in the subcutaneous MIA PaCa-2 model (Figures 5E,F). In mice bearing subcutaneous MIA PaCa-2 tumors (Figure 5E), treatment with TG6002/5-FC administered via three intravenous injections at 1×10⁶ PFU resulted in modest but significant tumor growth inhibition compared to untreated controls (*p* < 0.05). Irinotecan alone significantly suppressed tumor growth (*p* < 0.001), although its effect waned after treatment cessation. Notably, the combination of TG6002/5-FC and irinotecan produced the most pronounced and sustained antitumor response, significantly outperforming either monotherapy (*p* < 0.05). These findings suggest that the potent antitumor activity of irinotecan is further enhanced by TG6002/5-FC, with the combination showing highly significant efficacy compared to controls (*p* < 0.0005). In contrast, oxaliplatin alone, 5-FU alone, or the combination of oxaliplatin and 5-FU showed no therapeutic effect compared to untreated controls (Figure 5F). However, the combination of TG6002/5-FC and oxaliplatin significantly inhibited tumor growth relative to other groups (*p* < 0.01). Despite oxaliplatin’s lack of standalone efficacy in reducing MIA PaCa-2 tumor growth, its combination with TG6002/5-FC resulted in long-term tumor volume stabilization (Figure 5F).

### *Ex vivo* infection of patient-derived tumor tissue

**Figure 6:**
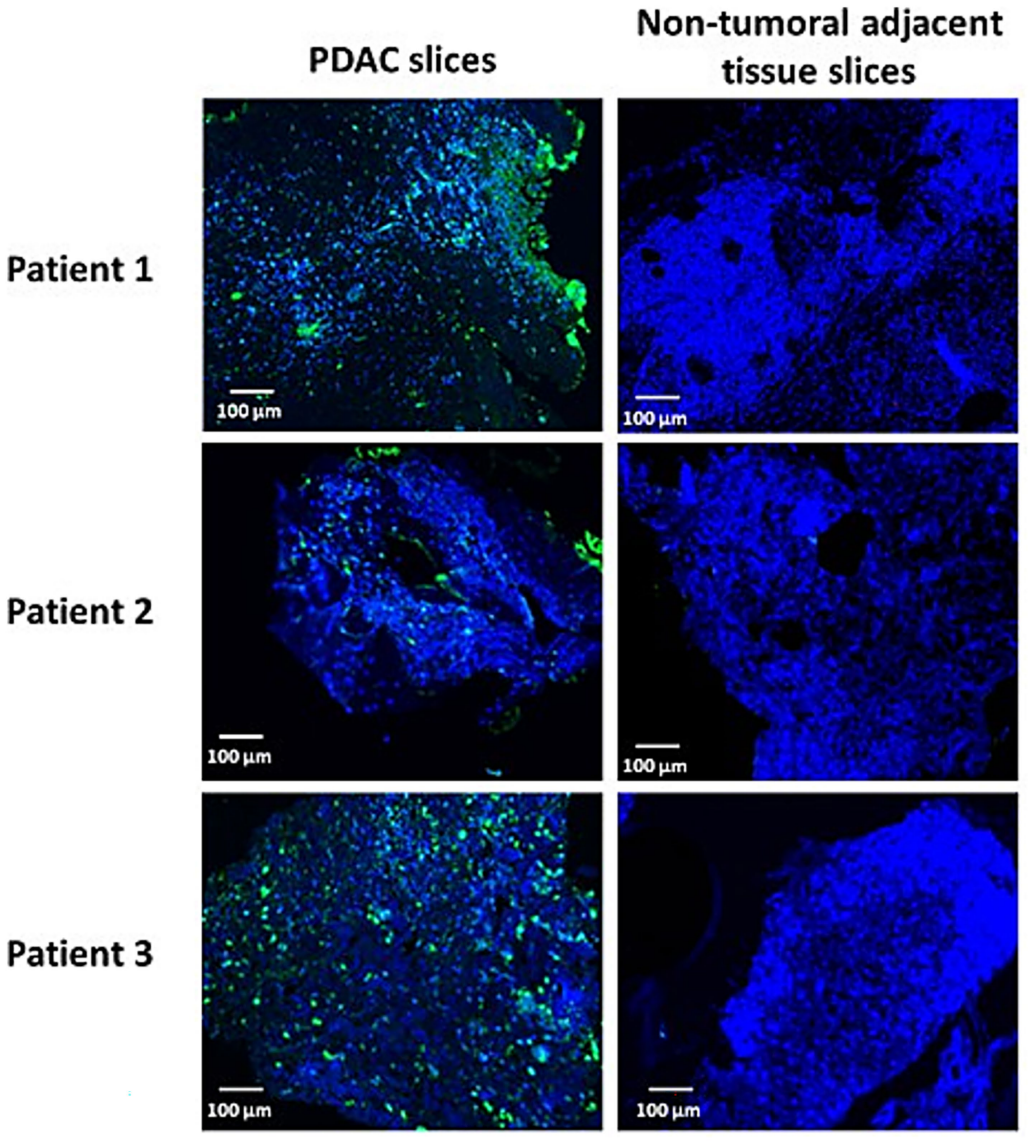
TG6002 infection and spreading in patient-derived PDAC slice cultures. Whole mount staining using Anti-VACV antibody (green) on PDAC and non-tumoral adjacent tissue slice cultures 3 days after infection with 1 × 10^6^ PFU of TG6002. DAPI (blue) was used as nuclear counterstain.

We investigated the susceptibility of PDAC biopsies and corresponding non-tumoral adjacent tissues to TG6002 infection. The identity of the surgical samples, PDAC and non-tumoral tissues, was confirmed through histopathological analysis by certified pathologists. Fresh biopsy samples were promptly transported to the laboratory, sectioned using a vibratome, and cultured with TG6002. After 3 days of incubation, the samples were fixed, embedded, and analyzed for TG6002 distribution using immunohistochemistry (Figure 6). In samples from three patients, PDAC slices exhibited multiple VACV-positive patches, indicating a successful TG6002 infection. In contrast, no VACV signal was detected in the non-tumoral adjacent tissues from the same patients (Figure 6). These findings suggest that the TK- and RR-deleted VACV selectively replicates in PDAC tissues, demonstrating its potential for tumor-specific targeting in PDAC patients.

## Discussion

PDAC remains one of the most aggressive and treatment-resistant cancers, with limited effective therapeutic options. This unmet medical need underscores the urgency for innovative treatments, particularly for patients diagnosed at various stages of the disease. Oncolytic viruses have emerged as a promising area of research. In this study, we evaluated TG6002, an oncolytic VACV expressing the suicide gene *FCU1*, as a potential therapeutic agent for PDAC.

Recent RNA expression analyses of PDAC cohorts have identified two major transcriptional subtypes with distinct biological and clinical characteristics. The “Classical” or progenitor subtype exhibits epithelial marker expression and is associated with a favorable prognosis, whereas the “Basal-like” or quasi-mesenchymal subtype is marked by mesenchymal features and aggressive clinical behavior (Moffitt et al., 2015; Bailey et al., 2016; Collisson et al., 2019). Notably, a recent study assessing five OV families (herpes simplex virus, adenovirus, measles virus, reovirus, and parvovirus) across 14 patient-derived PDAC cultures revealed subtype-specific sensitivities (Schafer et al., 2024). The progenitor subtype was resistant to herpes simplex virus, reovirus, and parvovirus, highlighting the influence of molecular subtypes on OV efficacy.

In our study, we tested TG6002 on five human PDAC cell lines representing epithelial (AsPc-1, BxPc-3, Capan-2) and mesenchymal (MIA PaCa-2, PANC-1) subtypes. Consistent with previous findings (Foloppe et al., 2019), TG6002 demonstrated robust infection, replication, and oncolytic efficiency across all tested PDAC cell lines, regardless of subtype. Many OVs, such as adenovirus, coxsackievirus or measles virus, require a specific receptor to enter the cell, which limits their tumor indication spectrum. For example, the expression of the primary infection receptor of adenovirus, the coxsackievirus-adenovirus receptor (CAR), is often decreased on pancreatic cancers (Pearson et al., 1999; Wesseling et al., 2001), making adenovirus less suitable for oncolytic virotherapy against PDAC. Unlike other OVs, VACV does not require a specific surface receptor. VACV is dependent only on glycoproteins that provide cell binding and membrane fusion to mediate entry, allowing it to reach of a wide range of tumor cells independently of receptor expression (Moss, 2006).

Measles virus, vesicular stomatitis virus but also to a lesser extent parvovirus and herpes simplex virus, are restricted by interferon-stimulated genes (ISGs) in PDAC (Schafer et al., 2024). In our study, VACV infectivity and killing of PDAC cells were independent of the type I IFN profile. TG6002 infection, replication and oncolysis were observed in cell lines displaying expression of IFN-stimulated antiviral genes (AsPC-1, Capan-2 and PANC-1; Moerdyk-Schauwecker et al., 2013). VACV has developed multiple mechanisms of immune evasion that allow it to replicate and spread widely throughout tumors. For example, VACV had evolved extracellular and intracellular mechanisms to block the immune response to IFN (Perdiguero and Esteban, 2009), the first response to viral infection, unlike RNA oncolytic viruses such as VSV or measles that were rapidly inhibited by IFN pathway activation (Murphy et al., 2012; Kurokawa et al., 2018). VACV encodes soluble interferon (IFN) receptors (B8R and B19R) that block the binding of IFNs to their cell-surface receptors (Alcami and Smith, 1995; Alcami et al., 2000). Moreover, the VACV E3L protein can mediate potent inhibitory activity against all human type I and type II IFNs, mainly through inhibition of the PKR pathway (Arsenio et al., 2008). Thus, the non-specific receptor independent entry and the development of many strategies to interfere with the IFN response make oncolytic VACV a versatile platform to address many tumor types and indications.

To assess TG6002’s relevance for PDAC, we employed advanced models, including 3D cell cultures and patient-derived tumor slices. It is now well established and reported in numerous publications that 3D culture is more representative of the TME and better suited to capture and demonstrate the treatment response in cancer. Indeed, spheroids appear to exhibit cell interactions, necrotic and hypoxic aspects that mimic the natural state of tumor patients (Vinci et al., 2012). Thus, the use of 3D cell culture is a mandatory way to predict the efficacy of a drug. PANC-1 spheroids represent a relevant 3D model for anti-pancreatic cancer drug screening (Yeon et al., 2013). PANC-1 spheroids showed expression of drug resistance-related molecules, including TGF-β1, CTGF, and MT1-MMP, and extracellular matrix proteins such as collagen type I, fibronectin, and laminin (Yeon et al., 2013). Moreover, PANC-1 cells grown as spheroids showed changes in stem cell populations and in expression levels of miRNAs that may play roles in chemoresistance (Yeon et al., 2013). In our study, oncolytic TG6002 replicated efficiently in PANC-1 spheroids, causing inhibition of 3D growth of spheroids, even at low doses, indicating good virus penetration and spreading of the virus. Complete deamination of 5-FC to 5-FU was also achieved in infected PANC-1 spheroids indicating good expression of the *FCU1* suicide gene in PDAC spheroids.

Patient-derived tumor slice cultures further validated TG6002’s efficacy. Tumor slice cultures preserve the original TME, and the use of tumor slice cultures has been demonstrated to be a valuable model for clinical and research studies (Kenerson et al., 2020). Several studies showed that PDAC slices from surgical biopsies maintain cellular integrity and heterogeneity (Misra et al., 2019; Weitz et al., 2021). Using a standardized protocol, we showed that TG6002 selectively infected PDAC slices while sparing adjacent non-tumoral tissue, underscoring its tumor specificity.

The therapeutic impact of TG6002 was evaluated in subcutaneous and orthotopic PDAC xenograft models. In the subcutaneous MIA PaCa-2 model, a single IT injection of TG6002 results in complete tumor shrinkage whatever the dose used. However, the late detection of PDAC in patients often leads to the presence of metastases. Consequently, an effective treatment for pancreatic cancer that can target both primary and metastatic tumors requires systemic injection. In the subcutaneous MIA PaCa-2 model, a single IV administration of TG6002 resulted in a weak inhibition of tumor growth, and the 5-FC treatment significantly improved the TG6002 antitumoral activity. We evaluated treatment efficacy using an orthotopic PDAC model, a more clinically relevant system that mimics the histopathological features and complexity of the PDAC microenvironment (Fernandez et al., 2023). This model offers significant advantages, including the ability to reproduce organ-specific conditions, tumor-host interactions, invasion, metastasis, and treatment responses (Hoffman, 2015). The orthotopic model demonstrated tumor-specific TG6002 localization and efficacy, highlighting its potential for systemic administration.

The TG6002/5-FC strategy offers several advantages over traditional 5-FU-based chemotherapy. The *FCU1* gene encodes a bifunctional enzyme that converts 5-FC to 5-FU and subsequently to 5-fluorouracil-monophosphate (5-FUMP), bypassing common chemoresistance mechanisms (Erbs et al., 2000). The rapid intracellular conversion of 5-FC to 5-FU and its diffusion into neighboring cells create a robust bystander effect, effectively targeting cancer cells beyond the initially infected ones. This enhances the overall therapeutic outcome (Erbs et al., 2000). The delivery of FCU1 to tumors via viral vectors has previously been explored using replication-deficient and oncolytic adenoviruses (Erbs et al., 2000; Dias et al., 2010; Quirin et al., 2011; Badder et al., 2024), as well as replication-deficient and oncolytic vaccinia virus (Erbs et al., 2008; Foloppe et al., 2008; Kurosaki et al., 2021), cowpox virus (Ricordel et al., 2017), raccoonpox virus (Ricordel et al., 2018), and measles virus (Kaufmann et al., 2013). These studies demonstrated localized delivery of 5-FU via FCU1 both *in vitro* and *in vivo*, with therapeutic efficacy enhanced when combined with 5-FC. Moreover, the use of the bifunctional FCU1 protein, instead of yeast or bacterial cytosine deaminase, significantly enhances the efficiency of converting 5-FC to 5-FU, giving FCU1 a clear advantage over earlier strategies that relied on cytosine deaminase/5-FC systems (Kievit et al., 1999; Erbs et al., 2000). Using 3D bioprinting technology, we recently examined the impact of the extracellular matrix (ECM) on TG6002 efficiency in a 3D model comprising colorectal tumor cells, cancer-associated fibroblasts (CAF), and a bio-inspired proliferative bioink (composed of gelatin, alginate, and fibrinogen) (Marquette et al., 2024). This study revealed a limited ability of the virus to spread through the ECM. In this 3D bioprinted model, and in the presence of 5-FC, tumor spheroid mortality was attributed exclusively to 5-FU production. Due to its ability to passively diffuse through cell membranes and the ECM (bystander effect), 5-FU affected not only the infected spheroids but also neighboring tumor clusters (Marquette et al., 2024).

We explored TG6002’s interactions with standard PDAC chemotherapies, including gemcitabine, oxaliplatin, and irinotecan. In 1997, gemcitabine monotherapy became the standard of care for the palliative first-line treatment of PDAC, although only a modest but statistically significant overall survival (OS) benefit was demonstrated when compared with 5-FU (Burris et al., 1997). In our *in vitro* study, when combined simultaneously with gemcitabine, the replication rate of TG6002 in the PDAC cell lines was largely reduced and the antitumor capacity was inhibited compared to TG6002 or gemcitabine as monotherapy. As a terminal nucleoside analog, one of the multiple modes of action of gemcitabine is to block DNA replication by covalently binding to the ribonucleotide reductase subunit M1 (RRM1) resulting in reduced cellular deoxyribonucleotide (dNTP) pools (Pereira et al., 2004). We have previously demonstrated that inactivation of cellular RRM1 using siRNA significantly decreases TG6002 replication indicating that TG6002, due to deletion of the large subunit of viral RR, is highly dependent on cellular RR levels (Foloppe et al., 2019). Therefore, it is not surprising that inhibitors of RRM1, such as gemcitabine, induce a decrease in TG6002 replication. Other groups have described similar interactions between oncolytic vaccinia viruses and chemotherapeutic agents. JX594, another oncolytic vaccinia virus, was also shown to be inhibited *in vitro* by Sorafenib, a B-raf kinase and VEGF receptor inhibitor approved for hepatocellular carcinoma (HCC) (Heo et al., 2011). However, the same study showed that sequential administration of JX-594 and sorafenib induced anti-tumor activity, indicating the importance of administration sequence. Gemcitabine is a promising therapeutic agent for PDAC, and our findings underscore the importance of carefully optimizing the administration schedule of TG6002 in combination with gemcitabine. Given the inhibitory effect of gemcitabine on viral replication, future studies should focus on identifying the most effective dosing, timing, and sequencing strategies to maximize therapeutic synergy while preserving viral efficacy.

The analysis of virus-drug interactions using the Chou-Talalay method (Chou and Talalay, 1984) demonstrated that TG6002 synergizes with oxaliplatin and SN-38 (the active metabolite of irinotecan) across all tested cell lines. Consistent with previous studies (Ziauddin et al., 2010; Ottolino-Perry et al., 2015), the observed combined efficacy *in vitro* was not due to increased replication of the oncolytic VACV but rather to a significant enhancement of apoptosis in tumor cells. This synergistic effect, combining two chemotherapeutic agents with the TG6002 + 5-FC-based oncolytic vaccinia virus, was further validated in xenograft models. FOLFIRINOX, a regimen combining 5-FU, folinic acid, irinotecan, and oxaliplatin, is a standard first-line treatment for advanced PDAC (Zhang et al., 2021). It has been shown to improve survival in patients with metastatic pancreatic cancer compared to gemcitabine, though it is associated with increased toxicity (Conroy et al., 2011). The toxicity of the FOLFIRINOX regimen, primarily attributed to 5-FU (Mahaseth et al., 2013), limits its use to patients with excellent performance status (Conroy et al., 2013). Our previous studies demonstrated that the FCU1-armed oncolytic vaccinia virus restricts 5-FU production to the tumor site, thereby mitigating the adverse effects caused by systemic 5-FU exposure (Foloppe et al., 2019). In Phase I clinical trials evaluating TG6002 administered either intravenously or via intrahepatic artery in combination with oral 5-FC, the treatment exhibited a favorable safety profile (Moreno Garcia et al., 2022; West et al., 2025). No dose-limiting hepatotoxicity or significant hematological adverse events were reported. Liver function parameters remained within normal ranges across all dose levels, and hematological indices were stable throughout the study. Although peripheral immune activation was observed, it did not result in clinically relevant hematological toxicity (West et al., 2025). These findings support the overall tolerability of TG6002/5-FC and strongly advocate for the clinical development of TG6002 in combination with 5-FC rather than 5-FU, particularly alongside irinotecan and oxaliplatin. In our study, all *in vivo* data were obtained from immunodeficient mice. While this model allows for the evaluation of direct oncolytic effects, it lacks a functional immune system, which is known to play a crucial role in the antitumor activity of oncolytic viruses (Wang et al., 2022). This limitation affects the interpretability of the results and precludes the assessment of potential synergistic effects with immunotherapeutic agents such as immune checkpoint inhibitors (ICIs). Future studies should therefore be conducted in immunocompetent or humanized models to better capture the contribution of the immune system and to explore the therapeutic potential of combining TG6002 with immune checkpoint blockade in PDAC. PDAC remains one of the most lethal malignancies, largely due to its immunosuppressive TME and poor response to ICIs when used as monotherapy. Recent advances in oncolytic virotherapy have opened new avenues for enhancing the efficacy of ICIs in PDAC. OVs not only induce direct tumor cell lysis but also promote immunogenic cell death, leading to the release of tumor-associated antigens and the recruitment of immune effector cells such as CD8^+^ T cells and natural killer (NK) cells (Hwang et al., 2020). This immunological priming can convert “cold” tumors into “hot” ones, thereby sensitizing them to ICIs. A notable example is VG161, a genetically engineered HSV-1-based OV expressing IL-12, IL-15, and a PD-1/PD-L1 blocking peptide. Preclinical studies have demonstrated that VG161 significantly enhances immune cell infiltration and synergizes with PD-1 blockade to suppress tumor growth in murine models of PDAC (Shen et al., 2023). Building on these findings, a multicenter phase I/II clinical trial (NCT05162118) is currently evaluating the combination of VG161 and nivolumab in patients with advanced pancreatic cancer.

In conclusion, our study highlights TG6002 as a promising candidate for PDAC treatment. Its broad efficacy across PDAC subtypes and synergy with chemotherapy drugs position it as a versatile therapeutic agent. The TG6002/5-FC combination, with its tumor-selective 5-FU production, holds significant potential to improve patient outcomes while minimizing systemic toxicity. Further clinical investigation is warranted to optimize TG6002’s integration into PDAC treatment paradigms.

## Data Availability

The original contributions presented in the study are included in the article/supplementary material, further inquiries can be directed to the corresponding author/s.

## References

[ref1] Alcami A. Smith G. L. (1995). Vaccinia, cowpox, and camelpox viruses encode soluble gamma interferon receptors with novel broad species specificity. J. Virol. 69, 4633–4639. doi: 10.1128/JVI.69.8.4633-4639.1995, PMID: 7609027 PMC189264

[ref2] Alcami A. Symons J. A. Smith G. L. (2000). The vaccinia virus soluble alpha/beta interferon (IFN) receptor binds to the cell surface and protects cells from the antiviral effects of IFN. J. Virol. 74, 11230–11239. doi: 10.1128/jvi.74.23.11230-11239.2000, PMID: 11070021 PMC113220

[ref3] Arsenio J. Deschambault Y. Cao J. (2008). Antagonizing activity of vaccinia virus E3L against human interferons in Huh7 cells. Virology 377, 124–132. doi: 10.1016/j.virol.2008.04.014 18502465

[ref4] Badder L. M. Davies J. A. Meniel V. S. Maruskova M. Salvador-Barbero B. Bayliss R. J.. (2024). The alphavbeta6 integrin specific virotherapy, Ad5(NULL)-A20.FCU1, selectively delivers potent "in-tumour" chemotherapy to pancreatic ductal adenocarcinoma. Br. J. Cancer 131, 1694–1706. doi: 10.1038/s41416-024-02869-3, PMID: 39369056 PMC11555051

[ref5] Bailey P. Chang D. K. Nones K. Johns A. L. Patch A. M. Gingras M. C.. (2016). Genomic analyses identify molecular subtypes of pancreatic cancer. Nature 531, 47–52. doi: 10.1038/nature16965, PMID: 26909576

[ref9001] Bastogne T. Samson A. Vallois P. Wantz-Mezieres S. Pinel S. Bechet D.. (2010). Phenomenological modeling of tumor diameter growth based on a mixed effects model. J. Theor. Biol. 262, 544–552. doi: 10.1016/j.jtbi.2009.10.008 19835891

[ref6] Beguin J. Foloppe J. Maurey C. Laloy E. Hortelano J. Nourtier V.. (2020). Preclinical evaluation of the oncolytic vaccinia virus TG6002 by translational research on canine breast Cancer. Mol. Ther. Oncolytics 19, 57–66. doi: 10.1016/j.omto.2020.08.020, PMID: 33072863 PMC7533293

[ref7] Burris H. A. Moore M. J. Andersen J. Green M. R. Rothenberg M. L. Modiano M. R.. (1997). Improvements in survival and clinical benefit with gemcitabine as first-line therapy for patients with advanced pancreas cancer: a randomized trial. J. Clin. Oncol. 15, 2403–2413. doi: 10.1200/JCO.1997.15.6.2403, PMID: 9196156

[ref9] Chou T. C. Talalay P. (1984). Quantitative analysis of dose-effect relationships: the combined effects of multiple drugs or enzyme inhibitors. Adv. Enzym. Regul. 22, 27–55. doi: 10.1016/0065-2571(84)90007-4, PMID: 6382953

[ref10] Collisson E. A. Bailey P. Chang D. K. Biankin A. V. (2019). Molecular subtypes of pancreatic cancer. Nat. Rev. Gastroenterol. Hepatol. 16, 207–220. doi: 10.1038/s41575-019-0109-y, PMID: 30718832

[ref11] Conroy T. Desseigne F. Ychou M. Bouche O. Guimbaud R. Becouarn Y.. (2011). FOLFIRINOX versus gemcitabine for metastatic pancreatic cancer. N. Engl. J. Med. 364, 1817–1825. doi: 10.1056/NEJMoa1011923, PMID: 21561347

[ref12] Conroy T. Gavoille C. Samalin E. Ychou M. Ducreux M. (2013). The role of the FOLFIRINOX regimen for advanced pancreatic cancer. Curr. Oncol. Rep. 15, 182–189. doi: 10.1007/s11912-012-0290-4, PMID: 23341367

[ref13] Dias J. D. Liikanen I. Guse K. Foloppe J. Sloniecka M. Diaconu I.. (2010). Targeted chemotherapy for head and neck cancer with a chimeric oncolytic adenovirus coding for bifunctional suicide protein FCU1. Clin. Cancer Res. 16, 2540–2549. doi: 10.1158/1078-0432.CCR-09-2974, PMID: 20388844

[ref14] Dijk F. Veenstra V. L. Soer E. C. Dings M. P. G. Zhao L. Halfwerk J. B.. (2020). Unsupervised class discovery in pancreatic ductal adenocarcinoma reveals cell-intrinsic mesenchymal features and high concordance between existing classification systems. Sci. Rep. 10:337. doi: 10.1038/s41598-019-56826-9, PMID: 31941932 PMC6962149

[ref15] Erbs P. Findeli A. Kintz J. Cordier P. Hoffmann C. Geist M.. (2008). Modified vaccinia virus Ankara as a vector for suicide gene therapy. Cancer Gene Ther. 15, 18–28. doi: 10.1038/sj.cgt.7701098, PMID: 17992203

[ref16] Erbs P. Regulier E. Kintz J. Leroy P. Poitevin Y. Exinger F.. (2000). In vivo cancer gene therapy by adenovirus-mediated transfer of a bifunctional yeast cytosine deaminase/uracil phosphoribosyltransferase fusion gene. Cancer Res. 60, 3813–3822, PMID: 10919655

[ref17] Fernandez J. L. Arbogen S. Sadeghinia M. J. Haram M. Snipstad S. Torp S. H.. (2023). A comparative analysis of Orthotopic and subcutaneous pancreatic tumour models: tumour microenvironment and drug delivery. Cancers (Basel) 15:415. doi: 10.3390/cancers15225415, PMID: 38001675 PMC10670202

[ref18] Foloppe J. Kempf J. Futin N. Kintz J. Cordier P. Pichon C.. (2019). The enhanced tumor specificity of TG6002, an armed oncolytic vaccinia virus deleted in two genes involved in nucleotide metabolism. Mol. Ther. Oncolytics 14, 1–14. doi: 10.1016/j.omto.2019.03.005, PMID: 31011628 PMC6461584

[ref19] Foloppe J. Kintz J. Futin N. Findeli A. Cordier P. Schlesinger Y.. (2008). Targeted delivery of a suicide gene to human colorectal tumors by a conditionally replicating vaccinia virus. Gene Ther. 15, 1361–1371. doi: 10.1038/gt.2008.82, PMID: 18480846

[ref20] Gallardo F. Schmitt D. Brandely R. Brua C. Silvestre N. Findeli A.. (2020). Fluorescent tagged vaccinia virus genome allows rapid and efficient measurement of oncolytic potential and discovery of oncolytic modulators. Biomedicine 8:543. doi: 10.3390/biomedicines8120543, PMID: 33256205 PMC7760631

[ref21] Haller S. D. Monaco M. L. Essani K. (2020). The present status of Immuno-oncolytic viruses in the treatment of pancreatic Cancer. Viruses 12:1318. doi: 10.3390/v12111318, PMID: 33213031 PMC7698570

[ref22] Heinrich B. Klein J. Delic M. Goepfert K. Engel V. Geberzahn L.. (2017). Immunogenicity of oncolytic vaccinia viruses JX-GFP and TG6002 in a human melanoma in vitro model: studying immunogenic cell death, dendritic cell maturation and interaction with cytotoxic T lymphocytes. Onco. Targets. Ther. 10, 2389–2401. doi: 10.2147/OTT.S126320, PMID: 28496337 PMC5422459

[ref23] Heo J. Breitbach C. J. Moon A. Kim C. W. Patt R. Kim M. K.. (2011). Sequential therapy with JX-594, a targeted oncolytic poxvirus, followed by sorafenib in hepatocellular carcinoma: preclinical and clinical demonstration of combination efficacy. Mol. Ther. 19, 1170–1179. doi: 10.1038/mt.2011.39, PMID: 21427706 PMC3129795

[ref24] Hoffman R. M. (2015). Patient-derived orthotopic xenografts: better mimic of metastasis than subcutaneous xenografts. Nat. Rev. Cancer 15, 451–452. doi: 10.1038/nrc3972, PMID: 26422835

[ref9002] Hwang J. K. Hong J. Yun C. O. (2020). Oncolytic Viruses and Immune Checkpoint Inhibitors: Preclinical Developments to Clinical Trials. Int. J. Mol. Sci. 21. doi: 10.3390/ijms21228627 PMC7697902 33207653

[ref25] Kaufmann J. K. Bossow S. Grossardt C. Sawall S. Kupsch J. Erbs P.. (2013). Chemovirotherapy of malignant melanoma with a targeted and armed oncolytic measles virus. J. Invest. Dermatol. 133, 1034–1042. doi: 10.1038/jid.2012.459, PMID: 23223133

[ref26] Kenerson H. L. Sullivan K. M. Seo Y. D. Stadeli K. M. Ussakli C. Yan X.. (2020). Tumor slice culture as a biologic surrogate of human cancer. Ann. Transl. Med. 8:114. doi: 10.21037/atm.2019.12.88, PMID: 32175407 PMC7049013

[ref27] Kieler M. Unseld M. Bianconi D. Schindl M. Kornek G. V. Scheithauer W.. (2020). Impact of new chemotherapy regimens on the treatment landscape and survival of locally advanced and metastatic pancreatic Cancer patients. J. Clin. Med. 9:648. doi: 10.3390/jcm9030648, PMID: 32121198 PMC7141274

[ref28] Kievit E. Bershad E. Ng E. Sethna P. Dev I. Lawrence T. S.. (1999). Superiority of yeast over bacterial cytosine deaminase for enzyme/prodrug gene therapy in colon cancer xenografts. Cancer Res. 59, 1417–1421. 10197605

[ref29] Kurokawa C. Iankov I. D. Anderson S. K. Aderca I. Leontovich A. A. Maurer M. J.. (2018). Constitutive interferon pathway activation in tumors as an efficacy determinant following oncolytic Virotherapy. J. Natl. Cancer Inst. 110, 1123–1132. doi: 10.1093/jnci/djy033, PMID: 29788332 PMC6186520

[ref30] Kurosaki H. Nakatake M. Sakamoto T. Kuwano N. Yamane M. Ishii K.. (2021). Anti-tumor effects of MAPK-dependent tumor-selective oncolytic vaccinia virus armed with CD/UPRT against pancreatic ductal adenocarcinoma in mice. Cells 10:985. doi: 10.3390/cells10050985, PMID: 33922406 PMC8145488

[ref31] Lambert A. Schwarz L. Borbath I. Henry A. Van Laethem J. L. Malka D.. (2019). An update on treatment options for pancreatic adenocarcinoma. Ther. Adv. Med. Oncol. 11:5568. doi: 10.1177/1758835919875568, PMID: 31598142 PMC6763942

[ref32] Mahaseth H. Brutcher E. Kauh J. Hawk N. Kim S. Chen Z.. (2013). Modified FOLFIRINOX regimen with improved safety and maintained efficacy in pancreatic adenocarcinoma. Pancreas 42, 1311–1315. doi: 10.1097/MPA.0b013e31829e2006, PMID: 24152956

[ref33] Marquette C. A. Petiot E. Spindler A. Ebel C. Nzepa M. Moreau B.. (2024). 3D bioprinted CRC model brings to light the replication necessity of an oncolytic vaccinia virus encoding FCU1 gene to exert an efficient anti-tumoral activity. Front. Oncol. 14:1384499. doi: 10.3389/fonc.2024.1384499, PMID: 39091906 PMC11292208

[ref34] Misra S. Moro C. F. Del Chiaro M. Pouso S. Sebestyen A. Lohr M.. (2019). Ex vivo organotypic culture system of precision-cut slices of human pancreatic ductal adenocarcinoma. Sci. Rep. 9:2133. doi: 10.1038/s41598-019-38603-w, PMID: 30765891 PMC6376017

[ref35] Moerdyk-Schauwecker M. Shah N. R. Murphy A. M. Hastie E. Mukherjee P. Grdzelishvili V. Z. (2013). Resistance of pancreatic cancer cells to oncolytic vesicular stomatitis virus: role of type I interferon signaling. Virology 436, 221–234. doi: 10.1016/j.virol.2012.11.014, PMID: 23246628 PMC3544977

[ref36] Moffitt R. A. Marayati R. Flate E. L. Volmar K. E. Loeza S. G. Hoadley K. A.. (2015). Virtual microdissection identifies distinct tumor- and stroma-specific subtypes of pancreatic ductal adenocarcinoma. Nat. Genet. 47, 1168–1178. doi: 10.1038/ng.3398, PMID: 26343385 PMC4912058

[ref9003] Moreno Garcia V. Cassier P. A. Doger de Spéville B. Calvo E. De Miguel M. J. Garcia-Carbonero R.. (2022). 392P Updated data of biodistribution and activity of oncolytic virus TG6002 after intravenous administration in patients with advanced gastrointestinal carcinomas. Annals. of Oncology. 33, S716. doi: 10.1016/j.annonc.2022.07.530

[ref37] Moss B. (2006). Poxvirus entry and membrane fusion. Virology 344, 48–54. doi: 10.1016/j.virol.2005.09.037, PMID: 16364735

[ref38] Murphy A. M. Besmer D. M. Moerdyk-Schauwecker M. Moestl N. Ornelles D. A. Mukherjee P.. (2012). Vesicular stomatitis virus as an oncolytic agent against pancreatic ductal adenocarcinoma. J. Virol. 86, 3073–3087. doi: 10.1128/JVI.05640-11, PMID: 22238308 PMC3302313

[ref39] Ottolino-Perry K. Acuna S. A. Angarita F. A. Sellers C. Zerhouni S. Tang N.. (2015). Oncolytic vaccinia virus synergizes with irinotecan in colorectal cancer. Mol. Oncol. 9, 1539–1552. doi: 10.1016/j.molonc.2015.04.009, PMID: 26004084 PMC5528789

[ref40] Park W. Chawla A. O'Reilly E. M. (2021). A review of pancreatic cancer-reply. JAMA 326, 2436–2437. doi: 10.1001/jama.2021.20068 34932080

[ref41] Pearson A. S. Koch P. E. Atkinson N. Xiong M. Finberg R. W. Roth J. A.. (1999). Factors limiting adenovirus-mediated gene transfer into human lung and pancreatic cancer cell lines. Clin. Cancer Res. 5, 4208–4213, PMID: 10632362

[ref42] Perdiguero B. Esteban M. (2009). The interferon system and vaccinia virus evasion mechanisms. J. Interf. Cytokine Res. 29, 581–598. doi: 10.1089/jir.2009.0073, PMID: 19708815

[ref43] Pereira S. Fernandes P. A. Ramos M. J. (2004). Mechanism for ribonucleotide reductase inactivation by the anticancer drug gemcitabine. J. Comput. Chem. 25, 1286–1294. doi: 10.1002/jcc.20054, PMID: 15139041

[ref44] Quirin C. Rohmer S. Fernandez-Ulibarri I. Behr M. Hesse A. Engelhardt S.. (2011). Selectivity and efficiency of late transgene expression by transcriptionally targeted oncolytic adenoviruses are dependent on the transgene insertion strategy. Hum. Gene Ther. 22, 389–404. doi: 10.1089/hum.2010.100, PMID: 20939692 PMC3073076

[ref45] Ricordel M. Foloppe J. Pichon C. Findeli A. Tosch C. Cordier P.. (2018). Oncolytic properties of non-vaccinia poxviruses. Oncotarget 9, 35891–35906. doi: 10.18632/oncotarget.26288, PMID: 30542506 PMC6267605

[ref46] Ricordel M. Foloppe J. Pichon C. Sfrontato N. Antoine D. Tosch C.. (2017). Cowpox virus: a new and armed oncolytic poxvirus. Mol. Ther. Oncolytics 7, 1–11. doi: 10.1016/j.omto.2017.08.003, PMID: 28951885 PMC5607123

[ref47] Schafer T. E. Knol L. I. Haas F. V. Hartley A. Pernickel S. C. S. Jady A.. (2024). Biomarker screen for efficacy of oncolytic virotherapy in patient-derived pancreatic cancer cultures. EBioMedicine 105:105219. doi: 10.1016/j.ebiom.2024.105219, PMID: 38941955 PMC11260584

[ref9004] Shen Y. Song W. Lin D. Zhang X. Wang M. Li Y.. (2023). VG161 activates systemic antitumor immunity in pancreatic cancer models as a novel oncolytic herpesvirus expressing multiple immunomodulatory transgenes. J. Med. Virol. 95:e28108. doi: 10.1002/jmv.28108 36042555 PMC10087349

[ref48] Siegel R. L. Miller K. D. Jemal A. (2015). Cancer statistics, 2015. CA Cancer J. Clin. 65, 5–29. doi: 10.3322/caac.21254 25559415

[ref49] Strobel O. Lorenz P. Hinz U. Gaida M. Konig A. K. Hank T.. (2022). Actual five-year survival after upfront resection for pancreatic ductal adenocarcinoma: who beats the odds? Ann. Surg. 275, 962–971. doi: 10.1097/SLA.0000000000004147, PMID: 32649469

[ref50] Vinci M. Gowan S. Boxall F. Patterson L. Zimmermann M. Court W.. (2012). Advances in establishment and analysis of three-dimensional tumor spheroid-based functional assays for target validation and drug evaluation. BMC Biol. 10:29. doi: 10.1186/1741-7007-10-29, PMID: 22439642 PMC3349530

[ref51] Von Hoff D. D. Ervin T. Arena F. P. Chiorean E. G. Infante J. Moore M.. (2013). Increased survival in pancreatic cancer with nab-paclitaxel plus gemcitabine. N. Engl. J. Med. 369, 1691–1703. doi: 10.1056/NEJMoa1304369, PMID: 24131140 PMC4631139

[ref9005] Wang L. Chard Dunmall L. S. Cheng Z. Wang Y. (2022). Remodeling the tumor microenvironment by oncolytic viruses: beyond oncolysis of tumor cells for cancer treatment. J. Immunother. Cancer. 10. doi: 10.1136/jitc-2021-004167 PMC9157365 35640930

[ref52] Weitz J. R. Tiriac H. Hurtado de Mendoza T. Wascher A. Lowy A. M. (2021). Using Organotypic tissue slices to investigate the microenvironment of pancreatic Cancer: Pharmacotyping and beyond. Cancers (Basel) 13:991. doi: 10.3390/cancers13194991, PMID: 34638476 PMC8507648

[ref53] Wesseling J. G. Bosma P. J. Krasnykh V. Kashentseva E. A. Blackwell J. L. Reynolds P. N.. (2001). Improved gene transfer efficiency to primary and established human pancreatic carcinoma target cells via epidermal growth factor receptor and integrin-targeted adenoviral vectors. Gene Ther. 8, 969–976. doi: 10.1038/sj.gt.3301473, PMID: 11438831

[ref54] West E. J. Sadoun A. Bendjama K. Erbs P. Smolenschi C. Cassier P. A.. (2025). A phase I clinical trial of intrahepatic artery delivery of TG6002 in combination with oral 5-fluorocytosine in patients with liver-dominant metastatic colorectal cancer. Clin. Cancer Res. 31, 1243–1256. doi: 10.1158/1078-0432.CCR-24-2498, PMID: 39785814 PMC11959272

[ref55] Yeon S. E. No da Y. Lee S. H. Nam S. W. Oh I. H. Lee J.. (2013). Application of concave microwells to pancreatic tumor spheroids enabling anticancer drug evaluation in a clinically relevant drug resistance model. PLoS One 8:e73345. doi: 10.1371/journal.pone.0073345 24039920 PMC3769301

[ref56] Zhang B. Zhou F. Hong J. Ng D. M. Yang T. Zhou X.. (2021). The role of FOLFIRINOX in metastatic pancreatic cancer: a meta-analysis. World J. Surg. Oncol. 19:182. doi: 10.1186/s12957-021-02291-6, PMID: 34154596 PMC8218408

[ref57] Zhu X. Fan C. Xiong Z. Chen M. Li Z. Tao T.. (2023). Development and application of oncolytic viruses as the nemesis of tumor cells. Front. Microbiol. 14:1188526. doi: 10.3389/fmicb.2023.1188526, PMID: 37440883 PMC10335770

[ref58] Ziauddin M. F. Guo Z. S. O'Malley M. E. Austin F. Popovic P. J. Kavanagh M. A.. (2010). TRAIL gene-armed oncolytic poxvirus and oxaliplatin can work synergistically against colorectal cancer. Gene Ther. 17, 550–559. doi: 10.1038/gt.2010.5, PMID: 20182517 PMC3250063

